# Digital Devices for Assessing Motor Functions in Mobility-Impaired and Healthy Populations: Systematic Literature Review

**DOI:** 10.2196/37683

**Published:** 2022-11-21

**Authors:** Christine C Guo, Patrizia Andrea Chiesa, Carl de Moor, Mir Sohail Fazeli, Thomas Schofield, Kimberly Hofer, Shibeshih Belachew, Alf Scotland

**Affiliations:** 1 Biogen Digital Health Biogen Inc Cambridge, MA United States; 2 Evidinno Outcomes Research Inc. Vancouver, BC Canada; 3 Biogen Digital Health International GmbH Biogen Inc Baar Switzerland

**Keywords:** motor function, medical devices, computers, handheld, smartwatch, smartphone, mobility, wearable electronic devices, Parkinson disease, Parkinsonian disorders, gait, mobile phone

## Abstract

**Background:**

With the advent of smart sensing technology, mobile and wearable devices can provide continuous and objective monitoring and assessment of motor function outcomes.

**Objective:**

We aimed to describe the existing scientific literature on wearable and mobile technologies that are being used or tested for assessing motor functions in mobility-impaired and healthy adults and to evaluate the degree to which these devices provide clinically valid measures of motor function in these populations.

**Methods:**

A systematic literature review was conducted by searching Embase, MEDLINE, CENTRAL (January 1, 2015, to June 24, 2020), the United States and European Union clinical trial registries, and the United States Food and Drug Administration website using predefined study selection criteria. Study selection, data extraction, and quality assessment were performed by 2 independent reviewers.

**Results:**

A total of 91 publications representing 87 unique studies were included. The most represented clinical conditions were Parkinson disease (n=51 studies), followed by stroke (n*=*5), Huntington disease (n=5), and multiple sclerosis (n=2). A total of 42 motion-detecting devices were identified, and the majority (n=27, 64%) were created for the purpose of health care–related data collection, although approximately 25% were personal electronic devices (eg, smartphones and watches) and 11% were entertainment consoles (eg, Microsoft Kinect or Xbox and Nintendo Wii). The primary motion outcomes were related to gait (n*=*30), gross motor movements (n*=*25), and fine motor movements (n*=*23). As a group, sensor-derived motion data showed a mean sensitivity of 0.83 (SD 7.27), a mean specificity of 0.84 (SD 15.40), a mean accuracy of 0.90 (SD 5.87) in discriminating between diseased individuals and healthy controls, and a mean Pearson *r* validity coefficient of 0.52 (SD 0.22) relative to clinical measures. We did not find significant differences in the degree of validity between in-laboratory and at-home sensor-based assessments nor between device class (ie, health care–related device, personal electronic devices, and entertainment consoles).

**Conclusions:**

Sensor-derived motion data can be leveraged to classify and quantify disease status for a variety of neurological conditions. However, most of the recent research on digital clinical measures is derived from proof-of-concept studies with considerable variation in methodological approaches, and much of the reviewed literature has focused on clinical validation, with less than one-quarter of the studies performing analytical validation. Overall, future research is crucially needed to further consolidate that sensor-derived motion data may lead to the development of robust and transformative digital measurements intended to predict, diagnose, and quantify neurological disease state and its longitudinal change.

## Introduction

### Background

Patient care is changing with the dawn of smart sensing technology. Mobile and wearable devices can provide continuous as well as objective monitoring and assessment of many health outcomes [1]. Until recently, outcomes that represent various motor functions (ie, any movement of the entire body or part of the body that is controlled by motor neuron activity) have typically been measured by patient reports (eg, number of falls) or physician assessment (eg, gait abnormalities). Physician assessments are based on very brief observations in an office or clinic [2], whereas self-reported outcomes are subjective and often not as sensitive nor as supervised as in-clinic measures [3]. Finally, measurements may vary between assessors depending on the level of training, familiarity, and experience [4,5].

Wearable technologies have recently emerged as a potential supplemental source of data on motor function. Such technologies could increase the objectivity and ease of assessment for motor functions during clinical trials and care while also allowing for a richer dimension of data to be captured. Real-world and continuous monitoring of patient motor functions through wearable and mobile sensors is increasingly being investigated in areas such as disease progression through motor fluctuations in Parkinson disease [6], detection of amyotrophic lateral sclerosis [7], and tremor activity in essential tremor [8].

Data from digital measurement solutions can enhance the quality of clinical trials, as illustrated by the acceptance of wearable device–measured stride velocity (95th percentile) by the European Medicines Agency (EMA) as an end point in Duchenne muscular dystrophy [9]. Given the implications these new data courses could have on the field, the current regulatory environment for mobile technologies is in flux [10]. US and European regulatory bodies are responding to this emerging opportunity by adapting their regulatory processes to these technological advances [11].

### Objectives

Previous reviews have described the characteristics of their patient samples and sensors involved in collecting motor function data [12-20]. However, they do not evaluate the degree of validity produced by such sensors. This review follows the terminology used in previous reviews [21,22] and differentiates between analytical validation (ie, the same motion behavior is measured by an independent source and compared with the sensor-derived motion behavior) and clinical validation (ie, a clinical characteristic or measure of interest is measured and compared with the sensor-derived motion behavior). Gaining insight into the current clinical validity and utility of the data captured by mobile and wearable sensing technologies is of utmost importance. So, the aim of this study was to describe the existing scientific literature on digital measurement solutions that are being used or tested for assessing motor functions in mobility-impaired and healthy adults and to evaluate the degree to which these tools provide clinically valid measures of motor function in these populations. Specifically, we aimed to answer the following research questions: (1) What types of digital devices exist that capture motor function in mobility-impaired and healthy populations? (2) In what types of studies and in what populations have these devices been evaluated? (3) What outcomes do these digital devices measure? (4) What types of technologies and algorithms are used to capture and store the data? (5) To what degree have these technologies and their output been validated using established and recognized criteria?

## Methods

### Literature Review

This review was conducted in accordance with the Cochrane Handbook for Systematic Reviews of Interventions [23], and reporting is based on the PRISMA (Preferred Reporting Items for Systematic Reviews and Meta-Analyses) guidelines [24]. We included clinical trials (randomized and nonrandomized) as well as observational studies (case-control, retrospective cohort, prospective cohort, and cross-sectional) that provided validity estimates from wearable or mobile technologies to assess motor functions in adults (aged ≥18 years). Studies published in English after 2015 were included to focus on the most advanced technologies that are being used to assess motor function.

Study eligibility criteria were defined using an adapted PICO (Population, Intervention, Comparator, Outcomes) framework. We applied criteria based on the technology instead of the intervention or comparator, as the research question focused on the validity of measurement and not treatment efficacy (Table S1 in Multimedia Appendix 1 [25-115]).

A systematic literature search was conducted (January 1, 2015, to June 24, 2020) in the MEDLINE, Embase, and CENTRAL databases. Searches of relevant conferences for the last 3 years (2018-2020) were conducted via Embase. Search strings are available in Tables S2-S6 in Multimedia Appendix 1. Gray literature searches were also conducted to capture studies from sources that were not included in the main literature databases, which included the US Food and Drug Administration website as well as the United States and European clinical trials registry databases for clinical trials which had reported results but were not published in peer-reviewed journals (for the years 2018-2020).

After duplicate removal, all titles and abstracts were screened for potential eligibility according to the prespecified PICO criteria, after which full-text articles were assessed using the same criteria. Study selection was performed by 2 independent reviewers, and disagreements were resolved through discussion. If no consensus could be achieved, a third researcher was consulted for arbitration.

A total of 2 independent reviewers extracted all relevant data from the final list of included studies. A reconciliation phase was again deployed to resolve any discrepancies between the reviewers, and a third reviewer intervened to resolve any remaining conflicts. The following data were extracted where available: (1) authors, year of publication, country, study setting, and follow-up period; (2) study design; (3) participant characteristics; (4) outcomes; (5) technology characteristics; and (6) validity outcomes. Motor function outcomes were manually sorted into categories by reviewers to facilitate summary where necessary.

### Study Quality

A total of 2 independent reviewers assessed the quality of the included studies using the ROBINS-E (Risk Of Bias In Nonrandomized Studies of Exposures) tool [116]. A third investigator intervened to reach consensus if there were any remaining unresolved discrepancies following reconciliation between the decisions of the 2 reviewers.

### Statistical Analyses

Effect size estimates were extracted from each study where reported, including standardized mean differences (ie, Cohen *d*), correlation coefficients (eg, Pearson *r*), sensitivity, specificity, accuracy, and area under the curve (AUC). In cases where studies provided none of these aforementioned effect size classes, effect sizes were calculated based on the information available in the manuscript using standard formulas [117,118]. To facilitate comparison across the studies, extracted effect sizes were converted to Pearson *r*–based effect size estimates where possible. This extraction and conversion process allows for studies to be directly compared via *r*-based effect sizes, estimates of sensitivity and specificity, and estimates of accuracy. The average effect sizes were calculated across all studies as well as by specific study and sample characteristics of interest. As *r* is bound by −1 and +1, *r*s were transformed into *Zr* using the procedure described by Fisher for analyses [119,120] and then back-transformed for reporting. Differences across groups in the magnitude of obtained effect sizes were tested using restricted information maximum likelihood derived SEs [117] using the inverse variance weight [121]. A random effects approach was taken, which includes in the denominator an extra variance component representing true variation in the population from which the included studies can be considered a random sample. A significance threshold of .05 was used to determine if values significantly differed between groups.

## Results

### Study Selection

**Figure 1:**
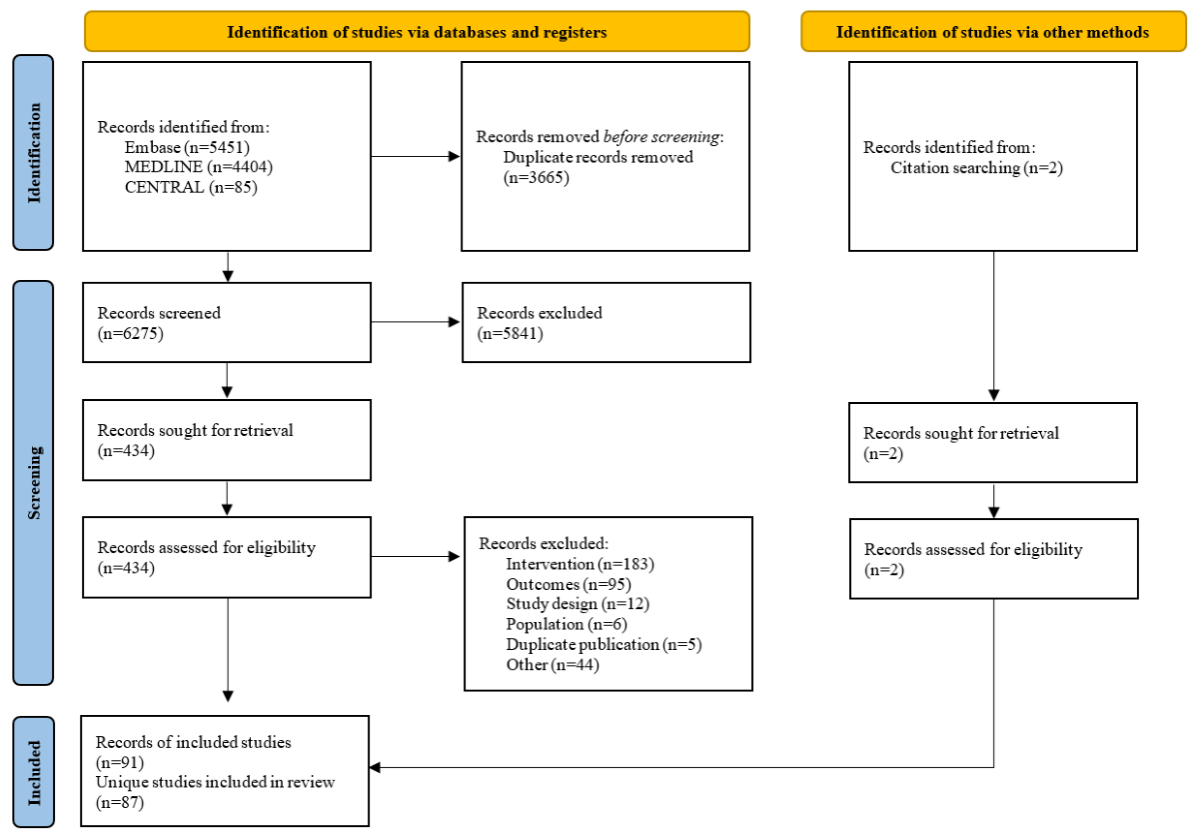
PRISMA (Preferred Reporting Items for Systematic Reviews and Meta-Analyses) flow diagram.

A total of 9940 abstracts were identified from the electronic databases, and 2 articles [25,26] were included from handsearching of a systematic review identified in our searches [122]. After the removal of duplicates and exclusion based on title and abstract screening, 436 records remained for the full-text screening. A list of the records excluded during full-text screening and the reason for exclusion are provided in Table S7 in Multimedia Appendix 1. A total of 91 publications describing 87 primary studies fulfilled all inclusion criteria (Figure 1).

### Study Characteristics and Data Collection

Across the 87 studies (n), the most common country settings reported were the United States (n=15) [27-41], United Kingdom (n=10) [42-53], Italy (n=5) [54-58], Spain (n=4) [59-62], South Korea (n=4) [63-66], Germany (n=3) [67-69], and Japan (n=3) [70-72]. At least 1 study was conducted in each of the following countries: Canada (n=2) [73,74], the Netherlands (n=2) [75,76], Portugal (n=2) [77,78], Sweden (n=1) [79,80], Taiwan (n=2) [81,82], Australia (n=1) [83], Brazil (n=1) [84], Demark (n=1) [85], France (n=1) [86], Israel (n=1) [87], Greece (n=1) [88,89], Lithuania (n=1) [90], Norway (n=1) [91], and United Arab Emirates (n=1) [92]. Of the remaining reporting studies, 6 were multinational [93-98]. Sample size ranged from 8 [33] to 1465 [94] (median 40.5 participants). A total of 7995 participants were enrolled in the included studies. Table S8 in Multimedia Appendix 1 presents the list of included publications as well as key study characteristics.

All 87 studies were observational in nature. Most studies (n*=*50) did not report whether the study was conducted in a single-center or multicenter setting. However, among those that did report, 20 and 17 studies were single center and multicenter, respectively. Approximately half of the included studies were conducted in a laboratory setting (n*=*42), 11 studies were home based, and 15 were a combination of a laboratory-based and home-based setting. The remaining 19 studies did not specify the study setting. The included studies were categorized into 2 follow-up types: cross-sectional (n*=*62) with a follow-up period of ≤1 week and longitudinal (n*=*25) in which participants were followed up for ≥1 week. Follow-up length of longitudinal studies ranged from 7 days [42,45,59,91,99] to 8 years [46]. A total of 30 studies reported the time allocated for data collection; in other words, the time needed to collect data in one session of data collection. In addition, 18 studies were able to capture their data in a session between 20 seconds [52,95] and 24 hours [71]. Moreover, 13 studies required their participant to use the device for multiple days for their collection period, which ranged from 2 [41,62] to 14 consecutive days [40]. This review follows the terminology used in previous reviews [21,22] for analytical validation (ie, the same motion behavior is measured by an independent source and compared with the sensor-derived motion behavior) and clinical validation (ie, a clinical characteristic or measure of interest is measured and compared with the sensor-derived motion behavior). Analytic validation was only performed in 21% (13/62) of cross-sectional studies and 4% (1/25) of longitudinal studies. Most of these studies performed clinical validation of sensor-based motion data. Studies applied a wide variety of technologies to capture motion outcomes. Motion data were captured by ≥30 different devices, including novel wearables (18/42, 43% devices), smartphone or smart watch (13/42, 31%), mass market digital technology (7/42, 17%), other digital technology (eg, PC; 3/42, 7%), and mass market wearables (1/42, 2%). Approximately 1 in 5 studies included a mass market device.

**Figure 2:**
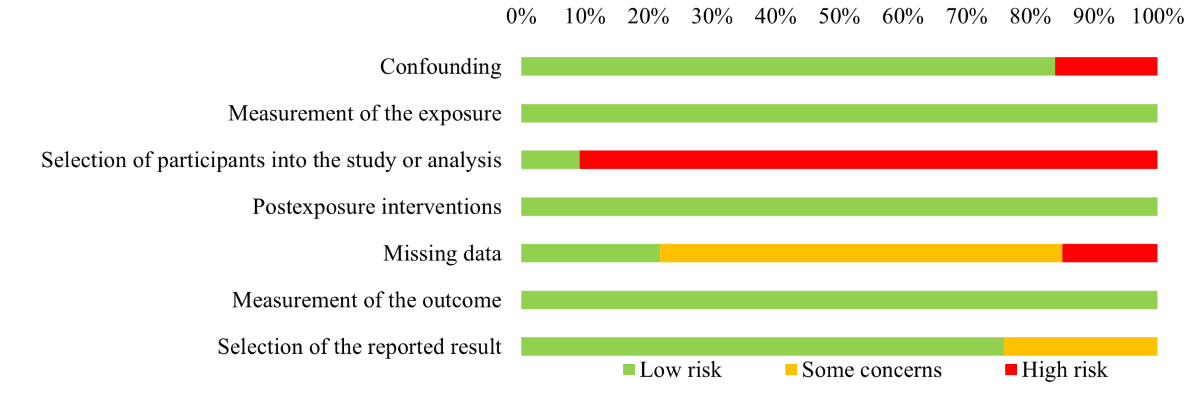
Distribution of study quality across included studies.

In terms of quality, studies were generally low to moderate risk of bias (Figure 2; Table S9 in Multimedia Appendix 1). Less than 20% (14/42) of studies did not show that groups were balanced in terms of key baseline characteristics and were considered high risk for confounding. The risk of bias arising from measurement of the exposure was most often low because exposures were generally whether the patient had a disease or was healthy, and misclassifications were next to nonexistent. For the domain of selection of participants into the study, studies were often high risk of bias. Disease diagnosis (ie, the exposure) did not generally coincide with the start of follow-up, and the diseases being studied could fluctuate over time. Many of the studies relied on volunteers to participate in the study, and this may have led to participants entering the study if they were in a particularly good or bad disease state (eg, Parkinson disease has *on* and *off* states). Furthermore, no corrections that may have alleviated selection biases in the analysis were conducted. Studies were generally low risk with regard to the domain concerned with the risk of bias owing to postexposure interventions. By design, the included studies did not administer interventions to alleviate the effects of exposures, and therefore, bias was not a concern. Regarding missing data, this was not often accounted for, leading to high risk of bias in that domain. However, studies were generally low risk of bias for measurement of outcomes, as motor function outcomes were assessed objectively and similarly across groups. Finally, over half of the studies were rated low risk for selection of the reported result.

### Concepts of Interest and Context of Use

Approximately half of the included studies compared the association between sensor-derived motion data and a standardized clinical assessment across diverse disease conditions (n=44). Other studies compared mobility-impaired diseased participants to a healthy control group of participants with no mobility impairment (n=43). The most represented disease condition was, by far, Parkinson disease (n=51); stroke (n=5); Huntington disease (n=5); and depression, cognitive impairment, cerebral palsy, and multiple sclerosis (n=2 for each). All other disease groups were only represented in a single study.

Among the 67 studies that reported the mean age of participants, values ranged from 23.6 years [92] to 77.2 years [95] for mobility-impaired participants and from 19.5 years [29] to 78.9 years [87] for healthy participants. Control groups were generally well-matched by participant age and sex. Among the 71 studies that reported the proportion of males or females in their sample, the average percentage of the sample that were male ranged from 22.8% [62] to 100% [72,84] in mobility-impaired participants and from 11% [41] to 100% [84] in healthy participants. Studies with the largest sex imbalances were those addressing the less frequently studied disease states (ie, represented in only 1 or 2 studies). In contrast, Parkinson disease, Huntington disease, and stroke reflected a more balanced representation of females and males.

The primary motion outcomes were gait (n=30), gross motor movements (n=25), fine motor movements (n=23), motor symptom severity (n=9), bradykinesia (n=7), motor fluctuations (n=6), dyskinesia (n=5), balance control (n=5), postural stability (n=4), voice or speech impairments (n=3), facial expression impairments (n=1), and nocturnal movements (n=1). A summary of commonly reported outcomes by disease that the outcome was measured in is provided in Table 1.

The most common motions that participants were required to enact for sensor data collection across these studies were based on diverse active motor tasks: multimovement tasks (16/87, 18%) including balancing and reaction time during tests such as the Timed Up and Go, the Cognitive Dual Task Timed Up and Go, and the Manual Dual Task Timed Up and Go, unscripted daily activities (17/87, 20%), walking (10/87, 11%), tapping (9/87, 10%), and scripted activities of daily living (7/87, 8%). Less commonly used motions (<5% of studies) included several real-world tasks such as reaching, sit-to-stand motion, seated tremors, wrist pronation-supination tracing or pointing, typing, seated conversation, standing, and sleeping movement. Together, these motions were used to extract ≥75 distinct motion outcomes across the included studies. Most of these outcomes only appeared in one study and were only measured at a single sensor location in each study (per our inclusion criteria). One exception was walking cadence, with different studies measuring it using sensors worn at wrists, ankles, lower back, and chest and in the pants pocket. Additional exceptions were tremor, dyskinesia, and bradykinesia (each measured using sensors placed on the wrists or ankles).

**Table 1:** Summary of commonly reported outcomes by disease in which the outcome was investigated.

Disease and motor function outcome category		Motor function outcome
**Acquired brain injury**		
	Gross motor impairment or performance and upper body	Peak upper limb velocity [35] Upper limb velocity [35]
**Alzheimer disease**		
	Fine motor impairment or performance and continuous motion	Spiral tracing [82]
**Depressive tendencies**		
	Fine motor impairment or performance and discrete motion	Finger tap speed [92] Flight time [92] Hold time [92]
**Healthy participants**		
	Bradykinesia	Bradykinesia score [94,100]
	Dyskinesia	Dyskinesia score [100]
	Fine motor impairment or performance and continuous motion	Spiral tracing [82,90]
	Fine motor impairment or performance and discrete motion	Correct finger taps [25,83] Finger tap accuracy [38,101] Finger tap count [38,95,101] Finger tap duration [38,101] Finger tap interval [38,101] Finger tap reaction time [38,42,58] Finger tap rhythm [42,95] Finger tapping test [102] Flight time [83,88,103] Hold time [88]
	Gait	Joint velocity [77] Step cadence [69,75,81,99] Step count [40,41,44,74,104] Step length [44,46,81] Stride duration [44] Turning speed [26] Walking speed [41,69,81]
	Gross motor impairment or performance and lower body	Lower limb velocity [105]
	Gross motor impairment or performance and whole body	Joint velocity [106]
	Motor symptom severity	Rest tremor [102]
	Postural stability	Trunk acceleration [50]
**Huntington disease**		
	Cognitive impairment	Stroop Color and Word Test [96]
	Dyskinesia	Chorea score [96,107]
	Fine motor impairment or performance, discrete motion	Finger tap speed [96]
	Gait	Step cadence [99]
**Mild cognitive impairment**		
	Fine motor impairment or performance and continuous motion	Spiral tracing [82]
**Multiple sclerosis**		
	Fine motor impairment or performance and discrete motion	Finger tap count [25]
	Gait	Turning speed [26]
**Neurological disorders^a^**		
	Fine motor impairment or performance and continuous motion	Spiral tracing [90]
**Neuromuscular disorders^b^**		
	Gait	Step count [104]
**Parkinson disease**		
	Bradykinesia	Bradykinesia score [34,48,53,94,97,100,108]
	Cognitive impairment	Stroop Color and Word Test [83]
	Dyskinesia	Dyskinesia score [53,100] Finger tapping test [56]
	Fine motor impairment or performance and discrete motion	Correct finger taps [83,109] Finger tap accuracy [38,101] Finger tap count [38,95,101] Finger tap duration [38,101] Finger tap interval [38,101] Finger tap reaction time [38,42,49] Finger tap rhythm [42,95] Finger tapping test [102] Flight time [88,103,110] Hold time [88]
	Gait	Freezing of gait [49,54,61,64,93,111,112] Step cadence [75] Step count [31,40] Step length [44,46] Stride duration [44] Turning speed [97]
	Gross motor impairment or performance and upper body	Peak upper limb velocity [33]
	Gross motor impairment or performance and whole body	Joint velocity [106]
	Motor fluctuations	On or off state [34,60,62,68,98]
	Motor symptom severity	Rest tremor [49,102] Tremor test [34,48,97]
	Postural stability	Trunk acceleration [50]
**Rapid eye movement (REM) sleep behavior disorder**		
	Fine motor impairment or performance and discrete motion	Finger tap reaction time [42] Finger tap rhythm [42]
**Stroke**		
	Gait	Step cadence [81] Step count [41,74] Step length [81] Walking speed [41,81]
**Transthyretin familial amyloid polyneuropathy**		
	Gait	Lower limb velocity [78] Step length [78] Stride duration [78] Walking speed [78]
	Gross motor impairment or performance and upper body	Upper limb velocity [78]

^a^Including Parkinson disease, Huntington disease, early dementia, cerebral palsy, and poststroke.

^b^Including Duchenne muscular dystrophy, limb-girdle muscular dystrophy, and spinal muscular atrophy.

### Data Processing and Analysis

**Figure 3:**
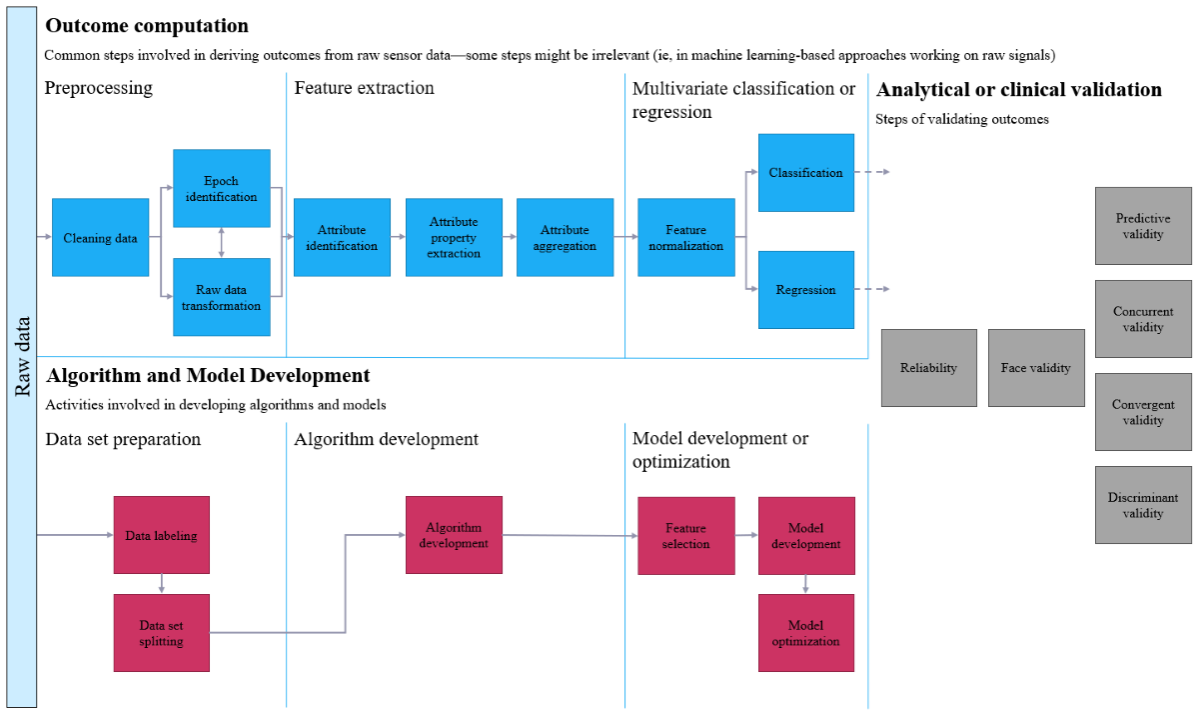
Flowchart of the process of converting raw data to validity coefficients.

The process through which these researchers converted their raw data to validity coefficients is illustrated in Figure 3. On collection of the raw data, 2 parallel processes were typically seen: outcome computation and algorithm or model development. Following the completion of these 2 processes, the model was subjected to either analytical or clinical validation.

### Outcome Preparation

In ≥90% of the studies, the raw data were first preprocessed before feature processing engineering and analyses. One preprocessing step frequently seen among these studies was the splitting of raw data into temporal epochs or slices. This was done because training an algorithm to detect movement features across long periods greatly reduced the algorithm’s validity. Data were trimmed by temporal position (eg, the beginning and ending of the motion recording) or based on extreme values (eg, outliers >4 SDs from the mean). Raw data were subjected to some form of standardization or transformation in ≥90% of the studies.

Although algorithm training (eg, feature selection and threshold determination) typically occurred using data across all participants, several studies took the approach of building the feature detection algorithm using data across all participants but then allowing each participant to vary in latter stages such as feature selection or determining thresholds [34,54,63,68]. Validity estimates from this smaller group of studies were similar in magnitude to those studies that applied the same features and thresholds to the classification of all participants.

Researchers have to decide which of the hundreds of identified candidate features to treat as a signal (by retaining them in the model) and which to dismiss as mostly noise (by excluding them from the model). Relatively few studies clearly described whether they moved all detected features to the next analytic stage (feature selection), but some studies compared prediction based on all extracted features to prediction based on top-performing features [42,49]. These studies reported that the inclusion of additional features did not guarantee a meaningful increase in algorithm performance or validity. One study using smartphones to assess Parkinson disease symptoms found AUC values >0.90 for 998 detected features, with a drop to 0.75 when based on the top 30 features [49]. A second study of participants with Parkinson disease concluded, “Accuracies obtained using the 30 most salient features were broadly comparable with the corresponding sensitivity and specificity values obtained using all 998 features” [42].

### Algorithm or Model Development

The included studies showed no clear preference regarding algorithms for feature selection or classification, but the 2 most frequently applied approaches were support vector machines (12/87, 14%) and random forests (4/87, 5%). Authors of these studies were sensitive to the complications of trying to train a classification model with groups of different sizes, as most of the comparative studies included in this review include approximately equal sizes of participants with a disease or disorder and healthy controls.

No consistent pattern emerged from within-study comparisons of feature selection algorithms. A wrist-based sensor was able to detect upper limb movement among participants with pre-Parkinson disease best when using random forests relative to support vector machines and naïve Bayes [55]. A smartphone app testing motor impairment found that both neural networks and boosting outperformed support vector machines and Fisher linear discriminant analysis [90]. Not all motions required feature selection across studies (several needed only to define logic rules to estimate movement angles using geometry), and some studies used proprietary algorithms that were not described in detail. One study that studied freezing of gait among participants with Parkinson disease using a smartphone app found neural networks performed better than other bagging algorithms, including random forest, multilayer perception, decision tree, support vector machine, and naïve Bayes [64]. Another study on motor symptoms among participants with Parkinson disease using ankle-worn sensors found that support vector machines performed better than logistic regression and decision trees [80]. Using smartphone motion data to predict motor impairment among participants with Parkinson disease, another study found that random forests based on Ridge regression outperformed those based on Lasso, or Gini impurity, and that linear support vector machines outperformed logistic regression and boosting [103]. The sole consistent pattern that emerged was that supervised machine learning techniques performed better than unsupervised techniques (eg, naïve Bayes).

### Analytical and Clinical Validation

The most common validity criterion was clinical condition (37/87, 43%), which was used in many of these studies to establish known-group construct discriminant validity of sensor-derived motion data by comparing participants with a diseased condition to healthy controls (Table S10 in Multimedia Appendix 1). The second most common validity criterion was the clinical validity established by assessing the convergence or concurrence with traditional standardized clinical assessments (30/87, 34%; eg, Wolf Motor Function Test and Unified Parkinson Disease Rating Scale). Other criteria were clinician ratings (7/87, 8%), research device (9/87, 10%), treatment status (3/87, 3%), and patient-reported outcome (1/87, 1%). Longitudinal studies were more likely to use nonsupervised assessments, whereas cross-sectional studies were more likely to use clinician-supervised assessments.

**Figure 4:**
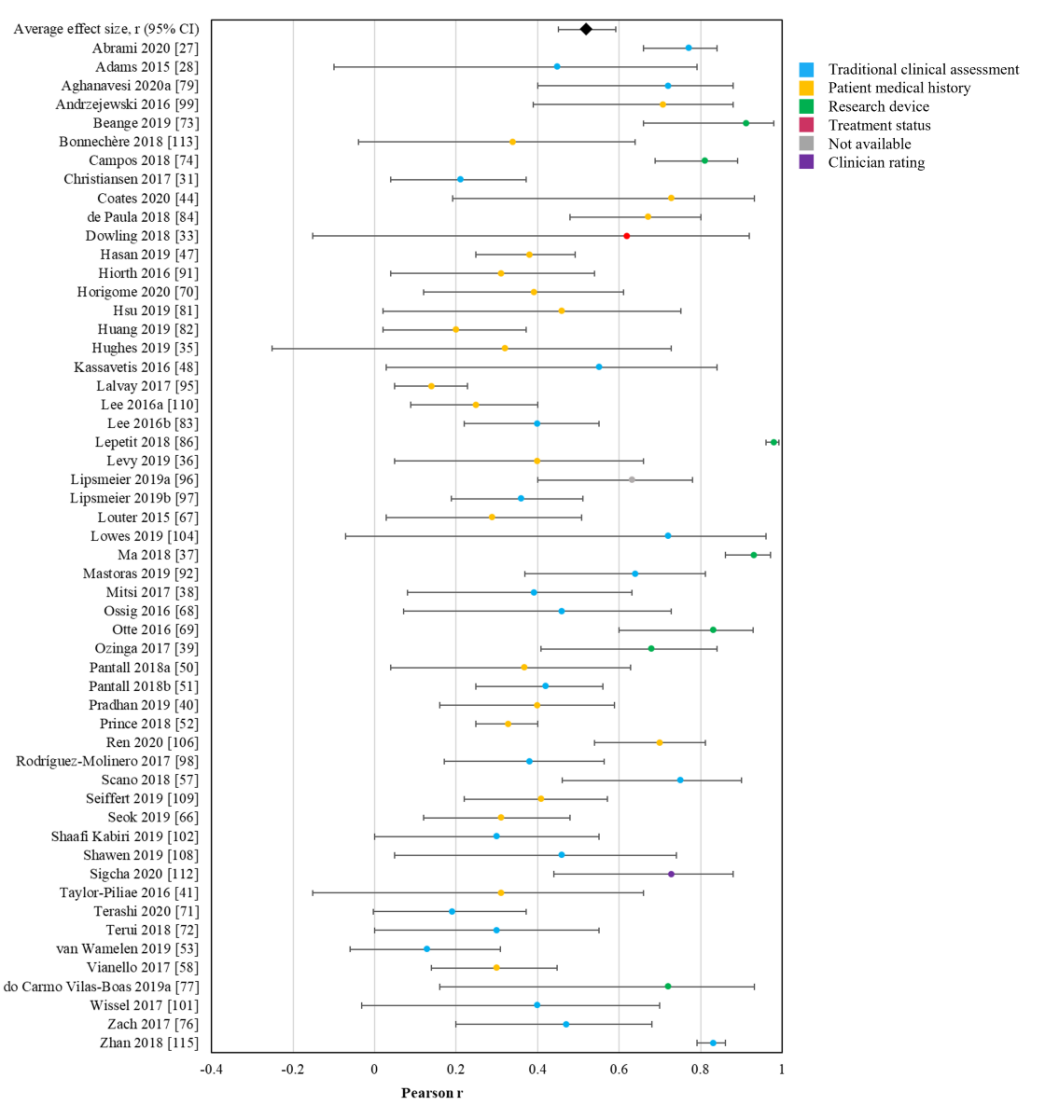
Forest plot of the validity of sensor-derived digital measurements of motor function. Middle points represent the point estimate effect size Pearson r, and the surrounding bars represent 95% CI. Colors indicate the type of validity criteria used.

Across studies, motion data from the sensors identified showed an average Pearson *r* clinical validity coefficient of 0.52 (Figure 4 [27,28,31,35-41,44,47,48,50-53,57,58,66-74,76,77,80-84,86, 91,92,95-99,101,102,104,106,108-110,112,113,115]). Among the studies that did not provide sufficient information to calculate a Pearson *r*, the average validity was 0.83 (sensitivity), 0.84 (specificity), and 0.90 (accuracy). These values could be interpreted as very good [123]. The magnitude of validity coefficients did not vary (*P*=.10) between health care–related devices (mean *r*=.47), personal electronic devices (mean *r*=.44), and entertainment consoles (mean *r*=.63). Validity coefficients for motor function generated by healthy adults were higher than those generated by participants with a disease state or impairment (*z* score 3.19; *P*=.001). The only statistical decision that consistently predicted higher validity coefficients was the decision to trim observations during the preprocessing stage based on value (ie, outliers; *z* score 2.10; *P*=.04). There was no difference in validity coefficients across trimming observations based on temporal placement, transforming data, standardizing data, or which feature detection and validation analyses were used. The funnel plot from these studies was asymmetrical in a manner consistent with bias toward higher coefficients (Figure S1 in Multimedia Appendix 1). The magnitude of validity coefficients did not significantly vary across the different device types (Table 2).

Taken as a whole, no consistent pattern emerged from within-study comparisons of the relative analytic validity of any specific motion signal. One study using Kinect found high Pearson *r* validity coefficients (*r*>0.50) for more than 40 distinct motion outcomes but very low validity coefficients for a handful including deflection range roll (measured in degrees), mean sway velocity roll (measured in degrees per second), and up-down deviation (measured in centimeters) [69]. A second study using Kinect found Pearson *r* validity coefficients above 0.50 for variables related to steps taken, distance, and speed but coefficients below 0.50 for variables related to angles (eg, trunk, hips, ankle, trunk, upper limb, and full body) [78]. A third study using a triaxial accelerometer worn on the waist found Pearson *r* validity coefficients above 0.50 for gait, arising from chair, body bradykinesia, hypokinesia, and overall posture and validity coefficients below 0.50 for rigidity of lower and upper extremities axial rigidity, postural stability, legs agility, and tremors in lower or upper extremities [98]. These numbers are in the same range as single items from widely established clinical tools [124-126]. As the validity coefficients for these single motions were moderate, it reinforces the need for future studies and clinical applications to include multiple validated motion signals for any screening or diagnostic tool to achieve adequate levels of composite test validity.

Regarding clinical validation, no clear within-study evidence emerged regarding the relative superiority or inferiority of motion data captured in laboratory settings versus data captured in home settings (Table 1). For example, 1 study comparing typing behavior of participants recently diagnosed with Parkinson disease to the typing behavior of healthy controls found AUC values of 0.76 (when administered at home) versus 0.83 (when administered in clinic) [59]. A second study comparing participants with Parkinson disease to healthy adults on motor function during an activities of daily living task found slightly higher accuracy, sensitivity, and specificity when the task was completed at home [87].

**Table 2:** Summary table of the between-study and within-study findings on the differences in the validity of sensor-derived measurements of motor function across various groups.

Are there differences in the validity of sensor-derived measures of motor function as captured	Between-study (ie, meta-analytic) findings	Within-study findings
Using mass market devices vs medical sensors?	No: digital technology vs mass market digital technologies (*P*=.22); mass market digital technology vs medical devices (*P*=.21); digital technology vs medical devices (*P*=.32)	Insufficient data to evaluate
At specific sensor locations?	No: wrist vs ankle (*P*=.73); wrist vs chest (*P*=.73); wrist vs hand (*P*=.54); wrist vs thigh (*P*=.59); wrist vs back (*P*=.63); wrist vs pocket (*P*=.78); wrist vs nonwearable (0.31) No: ankle vs chest (*P*=.46); ankle vs hand (*P*=.38); ankle vs thigh (*P*=.73); ankle vs waist (*P*=.60); ankle vs back (*P*=.49); ankle vs pocket (*P*=.65); ankle vs nonwearable (*P*=.58) No: chest vs hand (*P*=.30); chest vs thigh (*P*=.39); chest vs waist (*P*=.70); chest vs back (*P*=.82); chest vs pocket (*P*=.50); chest vs nonwearable (*P*=.89) No: hand vs thigh (*P*=.58); hand vs waist (*P*=.75); hand vs back (*P*=.78); hand vs pocket (*P*=.42); hand vs nonwearable (*P*=.53) No: thigh vs waist (*P*=.86); thigh vs back (*P*=.73); thigh vs pocket (*P*=.54); thigh vs nonwearable (*P*=.40) No: waist vs back (*P*=.87); waist vs pocket (*P*=.39); waist vs nonwearable (*P*=.24) No: back vs pocket (*P*=.45); back vs nonwearable (*P*=.48); pocket vs nonwearable (*P*=.50)	Insufficient data to evaluate
home vs in the laboratory?	No; *P*=.33	No; 1 study found AUC^a^ values of 0.76 (when administered at home) vs 0.83 (when administered in clinic) [59]. A second study found slightly higher accuracy, sensitivity, and specificity when the task was completed at home [87].
In longitudinal vs cross-sectional studies?	No; *P*=.29	No; One study found high Pearson *r* validity coefficients (*r*>0.50) for over 40 distinct motion outcomes but very low validity coefficients for a handful, including deflection rage roll (measured in degrees), mean sway velocity roll (measured in degrees per second), and up-down deviation (measured in centimeters) [69]. A second study found Pearson *r* validity coefficients above 0.50 for variables related to steps taken, distance, and speed, but coefficients below 0.50 for variables related to angles (eg, trunk, hips, ankle, trunk, upper limb, and full body) [78]. A third study found Pearson *r* validity coefficients above 0.50 for gait, arising from chair, body bradykinesia, hypokinesia, and overall posture and validity coefficients below 0.50 for rigidity of lower and upper extremities axial rigidity, postural stability, legs agility, and tremors in lower or upper extremities [98].
In healthy vs motor impaired patients?	Yes; validity higher among healthy adults, z score 3.19, *P*=.001	Insufficient data to evaluate
Using different feature detection algorithms?	Insufficient data to evaluate	No; One study was able to detect movement best when using random forests relative to support vector machines and naïve Bayes [55]. A second study found that both neural networks and boosting outperformed support vector machines and Fisher linear discriminant analysis [90]. A third study found neural networks performed better than other bagging algorithms including random forest, multilayer perception, decision tree, support vector machine, and naïve Bayes [64]. A fourth study found support vector machines performed better than logistic regression and decision trees [80]. A fifth study found that random forests based on Ridge regression outperformed those based on Lasso, or Gini impurity, and that linear support vector machines outperformed logistic regression and boosting [103]. The sole consistent pattern that emerged was that supervised machine learning techniques performed better than unsupervised techniques (eg, naïve Bayes).
Using particular motion sensor signal types?	Insufficient data to evaluate	Insufficient data to evaluate
Using all vs a subset of features?	Insufficient data to evaluate	No; One study found AUC values >0.90 for 998 detected features, with a drop to 0.75 when based on the top 30 features [49]. A second study concluded “Accuracies obtained using the 30 most salient features were broadly comparable with the corresponding sensitivity and specificity values obtained using all 998 features” [42].
With the thresholds held constant across patients vs patient-specific thresholds?	No; *P*=.48	No; Although algorithm training typically occurred across a sample, several studies took the approach of starting the algorithm (feature detection) using data across all participants but then allowing each patient to vary in later stages such as feature selection or determining thresholds [34,54,63,68]. Validity estimates from this smaller group of studies were similar in magnitude to those studies that applied the same features and thresholds to the classification of all participants.
Using clinically supervised vs nonsupervised assessments of patient clinical status?	No; *P*=.16	Insufficient data to evaluate
With outliers trimmed vs retained in the feature detection stage?	Yes; trimming outliers is beneficial, z score 2.10, *P*=.04	Insufficient data to evaluate
With transformed data vs untransformed data?	No; *P*=.74	Insufficient data to evaluate
With standardized data vs unstandardized data?	No; *P*=.60	Insufficient data to evaluate

^a^AUC: area under the curve.

## Discussion

### Principal Findings

To our knowledge, this is the first systematic literature review to evaluate the degree to which wearable and mobile technologies provide clinically valid measures of motor function in mobility-impaired and healthy adults. The identified literature generally consisted of proof-of-concept studies, which aimed to pilot a device and assess whether it could validly measure motor functions. Consequently, most studies used a short follow-up period (<1 week) and had a total sample size of <50 participants. Unsurprisingly, many of the longitudinal studies prioritized nonsupervised measures. Even so, taken together, these studies provide a respectable evidence base supporting the potential these movement sensors have to inform clinical practice.

As the eligibility criteria for our review were inclusive in terms of population, we identified a large range of disease types, which were all but one (chronic obstructive pulmonary disease) nervous system condition (Table 1); however, the most common disease was Parkinson disease, with stroke and Huntington disease coming in a very distant second and third place. The strong focus on Parkinson disease in this literature may be because of its prevalence or perhaps because motor function symptoms are a major characteristic of Parkinson disease for diagnosis and prognosis assessment purposes, making Parkinson disease an ideal model disease for testing the use of mobile technologies [127]. However, it is most probably a mixture of these 2 hypotheses. Parkinson disease is also one of the few diseases with Food and Drug Administration–approved devices (eg, NexStride and Personal KinetiGraph), which assesses motor function to inform treatment decisions. The field would benefit from additional study of mobile technology–assessed motor function among other neurological diseases, including multiple sclerosis, spinal muscular atrophy, amyotrophic lateral sclerosis, and Alzheimer disease. In addition, future studies might consider the advantages of assessing digital devices per neurological impairment (such as difficulties in ambulation or upper limbs) rather than per disease.

Successful integration of wearable-based movement data into clinical tools requires both analytic validation and clinical validation. However, most of the reviewed literature compared wearable sensor-derived motion data to omnibus measures of functioning or disease progression (ie, clinical validation). More studies need to perform analytic validation by comparing wearable sensor-derived motion data to the same motions measured by another source (eg, observer assessment and motion-capture technology). Observed motions may be highly correlated with omnibus assessments of motor skills or disease status (ie, clinical validation), but the foundation of approval as a clinical end point can only be met if the motions identified using the sensor have been shown to be the exact motions that have been approved by the governing or regulatory body. Using as an example the EMA’s recent approval of 95% stride velocity as an approved secondary end point in Duchenne muscular dystrophy, appeal to the EMA’s approval of wearable sensor stride velocity data as an end point for a given study requires evidence that when the used algorithm claims to measure stride velocity (95th percentile), there be evidence that the algorithm has, in truth, measured stride velocity. Future research in this area should focus their attention on analytic validation.

There was considerable variation in methodological approaches. The review revealed one of the key reasons why this field may still show such inconsistency in analytic approach; it is still developing. Evidence of this is seen in which motion variables could be identified by the algorithms. Despite the hundreds of motion-derived outcome variables identified across these studies, not all theoretically meaningful motions could be recovered. One study of participants with Parkinson disease concluded, “Unfortunately, we failed to find parameters that reflected fatigue (decrement response) and hesitation (intertap irregularity), which are characteristics of motor dysfunction in Parkinson’s disease” [110]. Those authors offered that more precise definitions of fatigue and hesitation may be needed to recover them in clinical settings with a smartphone-based tapping test similar to the one used in that study. In addition, the motor functions viewed by some authors as theoretically relevant were occasionally overshadowed by nonmotor signals. The tendency for studies to report diminishing returns after a certain point for additional motion signals is statistically analogous to other clinical efforts to identify causal markers from a multitude of candidates, which revealed many initially flagged markers as spurious [128]. Future studies should include graphical displays to identify inflection points (similar to the scree plot in factor analysis or the elbow plot in latent class analysis) to help show where the statistical signal (or true score) from additional motions becomes outweighed by statistical noise.

The moderate to high validity coefficients reported in the identified literature may support the potential for sensor-derived motor function data from digital health technology tools to eventually contribute to screening, diagnosis, and monitoring of neurological diseases in particular. No significant differences in analytic or clinical validity estimates were found when comparing data generated by mass market devices (eg, smartphones, smartwatches, and Fitbits), game consoles (ie, Nintendo Wii and Microsoft Kinect or Xbox), and marketed motion sensors (eg, ActiGraph, ActivPAL, Axivity, Dynaport, KinetiSense, Opal devices, and PAMSys-X). Furthermore, the motion data provided by these technologies produced equivalent validity estimates in laboratory-based and home-based settings. This further supports the future potential for digital measurement solutions to provide clinically meaningful data and eventually become the gold standard for assessing motor behaviors. The degree and rate of application for motor function data from these devices to clinical practice will depend on how soon clear evidence bases are established for given sensor locations for given movements of interest.

Translation of these motor signals into clinical application is aided by demonstrating sufficient validity outside the scripted protocols of a controlled laboratory setting. The reviewed literature showed that scripted motion tasks were important when only a few minutes of motion data were to be captured. Furthermore, motion data from unscripted everyday living with longer data collection periods were also shown to be adequate and deemed complementary, as episodic scripted assessments of confined tasks might not capture the complex spectrum of potentially altered components of motor function in an unconstrained ecologic setting [129].

As a whole, the reviewed literature revealed several best practices as well as a few cautionary tales for mobile or wearable sensor-based movement data. Although cross-validation techniques all seek to counteract the inflation of validity coefficients that can occur during machine learning techniques, they can produce different results [42]. Despite these best practices, there remained indirect evidence of model overfitting in the form of some abnormally high validity coefficients in the final models (ie, specificity of 1.0, which is perfect) [130,131].

The reviewed literature also highlights areas to consider during the development of any clinical application. One illustration from this review is the critical role of thresholds [132], which require researchers to decide between manual versus automatic thresholds [133] and global versus person specific [134]. Leveraging the strengths of these modeling approaches while keeping them robust and flexible will be important to consider as they are scaled up to create clinical applications [132].

### Comparison With Previous Reviews

We identified a number of similar literature reviews during our study selection [12-20]. All identified reviews synthesized their evidence qualitatively, and none provided a quantitative synthesis of the validity of motion data generated from these sensors among patients with neurological conditions. Of the 9 identified reviews, 1 was narrative [16], whereas the remaining were systematic reviews. None of the systematic reviews focused on neurological disorders. Overall, 2 reviews focused specifically on swimming motions [12,13], 2 were focused on older adult patients with no specific disease [15,19], and 2 reviews focused on only upper [14] and lower limb movements [18]. Of the remaining 2 systematic reviews with similar objectives and scope to that of our own, the paper by Díaz et al [17] aimed to review the current literature on the use of wearable sensors in gait, balance, and range of motion analysis. Diseases of participants also varied across their 56 included studies and included a mix of neurological disorders (eg, Parkinson disease, Alzheimer disease, and multiple sclerosis), as well as stroke, amputees, and healthy participants. Similar to our own review, the authors found that most body-worn devices were complex to use and required strong experience in data analysis to interpret the collected information. In addition, the authors pointed out a need for further validation and improvements in sensor systems for them to be used as reliable and accurate clinical devices. A second systematic review conducted by Kristoffersson and Lindén [20] provided a qualitative synthesis of 73 published articles on wearable body sensors used for health monitoring. Similar to our review, the authors found that included studies were generally observational in design and small in sample size. These methodological considerations should be taken into account for future studies testing clinical devices for assessing motor function.

### Strengths and Limitations

One strength of this review is that it includes more studies than any other review of similar scope that we identified during our study selection process [12-20]. This review is unique relative to other reviews on this same topic because it summarizes the validity estimates across the included studies instead of simply describing the characteristics of the samples and sensors involved [15-20]. This provides an evaluation of the degree of validity produced by such sensors. An additional strength was that we identified several meaningful patterns in this literature (eg, an absence of consistency in analytic approaches, equivalent validity of motion data collected at home or in a laboratory, and higher validity coefficients for healthy adults), which can help guide future research in this area. A final strength of this review is that it addresses statistical issues in this field. Although most reviews in this research area are silent as to statistical concerns, the findings of this review are consistent with the small group of previous reviews, which have also noted the statistical challenges present in this literature [12-14].

A limitation of this review is insufficient statistical power to address several questions of interest because of the methodological inconsistency and resulting sparseness across studies. A second limitation of this review is that the literature showed some signs of potential bias, which could limit the trustworthiness of the aggregate effect sizes. Examples of potential bias identified during the study quality assessment were that few studies provided a clear description of whether data were available for all participants throughout the study, and no studies corrected for potential selection biases in their analyses. In addition, it is unclear whether the patterns seen in the funnel plot and elsewhere are evidence of publication bias, selective outcomes, or an artifact of the dominant analytic approaches in this field. Much of the reviewed literature has focused on clinical validation, with less than one-fourth of the studies performing analytical validation. As important as clinical validation is for establishing the clinical and real-world utility of sensor-derived motion data, more studies are needed that focus on the fundamental step of analytic validation. An additional limitation may be the fact that some diseases are not as prevalent or well-studied than others, which may have impacted their representation in our analyses. Finally, our review was restricted to publications available in the English language. Therefore, some technologies being investigated for motor function assessment in non–English-speaking countries may have been missed.

### Considerations for Future Research

Several questions we initially hoped to answer in this review could not be addressed because of lack of consistency across studies (eg, which technology or sensor is used, where the sensor is placed, which motions are required by participants, preprocessing steps, feature detection and selection algorithms, and number of motion features retained for the prediction algorithm). Even within studies examining the same disease state, there was limited consistency in these characteristics. As a result, we cannot say which movements and motion outcomes produce the most valid indicators of different neurological disease states, or what data preprocessing, feature processing engineering, and analysis should be considered best practices for converting raw sensor-derived motion data into meaningful digital measurements or biomarkers. It was notable that many of the most common movements from the larger clinical literature (eg, reaching, sit-to-stand, tracing, and pointing) appeared so infrequently in this literature. This lack of consistency in the literature could have affected the validity estimates [135-139], and the lack of harmonization across studies limits any inference about methodological or analytic decisions [140].

An earlier review described continuous monitoring using movement-detecting wearable sensors as a potential source of ground truth for motor function data, which were previously available only through participant self-reports [141]. On the basis of the reviewed literature, the field cannot yet provide this type of objective truth. An existing algorithm needs to be applied to multiple samples without additional adjustments or enhancements and show an aggregate performance that approximates the estimates provided by the studies included in this review. No analytic technique will solve this issue; the only true solution is replication attempts in new samples. Researchers should report how many of the detected features were moved to feature selection to give readers a sense of how many features were excluded, a sense of the parsimony of the resultant model, and an awareness of how likely it is that the model may have been overfit. Care must be taken to design the classification algorithm in a way that maximizes the likelihood that it can perform equally well in future samples. This priority needs to be evaluated at each stage of the analysis: data set preparation, preprocessing, feature extraction, algorithm development, model development or validation, and analytical or clinical validation.

### Conclusions

In conclusion, sensor-derived motion data can be leveraged to validly predict disease status for a variety of neurological conditions. Future research will elucidate to what extent sensor-derived motion data may yield robust and transformative digital measurements intended to quantify, diagnose, and predict neurological disease state and its longitudinal change.

## Appendix

Multimedia Appendix 1 Supplemental information on the methods and results of the systematic literature review.

## Abbreviations

AUC: area under the curve
EMA: European Medicines Agency
PICO: Population, Intervention, Comparator, Outcomes
PRISMA: Preferred Reporting Items for Systematic Reviews and Meta-Analyses
ROBINS-E: Risk Of Bias In Nonrandomized Studies of Exposures
