# Supplementary material for: Digital Devices for Assessing Motor Functions in Mobility-Impaired and Healthy Populations: Systematic Literature Review
Source: J Med Internet Res. 2022 Nov 21;24(11):e37683. doi: 10.2196/37683 (PMC9723979; doi:10.2196/37683)

## Review

# Digital Devices for Assessing Motor Functions in Mobility Impaired and Healthy Populations: A Systematic Literature Review

## Supplementary Materials

Table S1: PICO eligibility criteria.

| **PICO Item** | **Inclusion Criteria** | **Exclusion Criteria** |
| --- | --- | --- |
| **Population** | - Healthy or unhealthy adults (aged >18 years) | - Children (aged <18 years) - Studies not enrolling patients - Animal studies |
| **Intervention or Technology characteristic** | - Validated digital and/or wearable technologies used or being tested for assessing motor functions in healthy or unhealthy adults - Any other validated technology related to the use of digital and/or wearable devices used or being tested for assessing motor functions in healthy or unhealthy adults | - Non-validated technologies - Complex, multi-sensor devices that do not report data separately for each sensor - Devices that only capture motion data using a non-electronic-device-related tool, including:   - Professional assessments, timed tests, diagnostics machines - Devices for use in assisting or improving functional mobility without capturing motion data |
| **Comparators** | - Any or none | - Not applicable |
| **Outcomes** | - Motor function outcomes measured by digital and/or wearable technology, including by not limited to:   - Gross motor functions (eg, gait speed, stride length, gait symmetry, bradykinesia)   - Fine motor functions (eg, finger tapping speed, tracing accuracy)   - Oculomotor function outcomes (eg, eye movements, pupillary reflex, blink) | - Patient-reported outcomes (eg, questionnaires) |
| **Study design** | - Randomized controlled trials - Non-randomized clinical trials - Observational studies   - Case-control studies   - Retrospective cohort studies   - Prospective cohort studies   - Cross-sectional studies - Modelling studies | - Notes - Letters - Editorials - Comments - Case reports or case series - Proof-of-concept studies - Studies designed to demonstrate the functionality of the device - Cost or economic studies |
| **Additional Criteria (Limits)** | | |
| **Language** | - English | - Non-English publications |
| **Publication date limit** | - Records published in 2015 onwards | - Not applicable |

Table S2: Search strategy for Embase via OvidSP.

| **Database: Embase 1974 to 2020 June 23**  **Search executed: June 24, 2020** | | |
| --- | --- | --- |
| **#** | **String** | **Hits** |
| 1 | (wearable* or device* or sensor* or smart* or digital or mobile or acceleromet* or gyroscope* or (inertial adj2 measur* adj2 unit*) or "IMU" or magnetomet* or "compass" or "gps" or touchscreen* or (touch adj2 screen*) or camera* or "kinect" or "infrared").ti,ab,kw. | 1437343 |
| 2 | (motor adj2 (skill* or function* or symptom* or sign* or impair* or control* or fluctuat*)).ti,ab,kw. | 109825 |
| 3 | 1 and 2 | 20779 |
| 4 | (book or chapter or editorial or erratum or letter or note or short survey or tombstone or comment or practice-guideline or journal correspondence or posters or news or newspaper article or lectures or interview or historical article or handbooks or guidelines or guidebooks or essays or database or catalogs).pt. | 3175823 |
| 5 | (conference or conference abstract or conference review).pt. | 4583742 |
| 6 | limit 5 to yr="2018-current" | 799765 |
| 7 | 5 not 6 | 3783977 |
| 8 | (exp animal/ or nonhuman/) not exp human/ | 6370182 |
| 9 | or/4,7-8 | 12739464 |
| 10 | 3 not 9 | 13108 |
| 11 | limit 10 to english | 12295 |
| 12 | limit 11 to yr="2015-Current" | 5493 |
| 13 | remove duplicates from 12 | 5451 |

Table S3: Search strategy for MEDLINE® via OvidSP.

| **Database: Ovid MEDLINE(R) and Epub Ahead of Print, In-Process & Other Non-Indexed Citations, Daily and Versions(R) 1946 to June 23, 2020**  **Search executed: June 24, 2020** | | |
| --- | --- | --- |
| **#** | **String** | **Hits** |
| 1 | (wearable* or device* or sensor* or smart* or digital or mobile or acceleromet* or gyroscope* or (inertial adj2 measur* adj2 unit*) or "IMU" or magnetomet* or "compass" or "gps" or touchscreen* or (touch adj2 screen*) or camera* or "kinect" or "infrared").ti,ab,kw. | 1164764 |
| 2 | (motor adj2 (skill* or function* or symptom* or sign* or impair* or control* or fluctuat*)).ti,ab,kw. | 72467 |
| 3 | 1 and 2 | 13435 |
| 4 | (book or chapter or editorial or erratum or letter or note or short survey or tombstone or comment or practice-guideline or journal correspondence or posters or news or newspaper article or lectures or interview or historical article or handbooks or guidelines or guidebooks or essays or database or catalogs).pt. | 2446971 |
| 5 | (exp animal/ or nonhuman/) not exp human/ | 4710249 |
| 6 | 3 not (4 or 5) | 11022 |
| 7 | limit 6 to english | 10502 |
| 8 | limit 7 to yr="2015-current" | 4477 |
| 9 | remove duplicates from 8 | 4404 |

Table S4: Search strategy for CENTRAL via OvidSP.

| **Database: EBM Reviews - Cochrane Central Register of Controlled Trials May 2020**  **Search executed: June 23, 2020** | | |
| --- | --- | --- |
| **#** | **String** | **Hits** |
| 1 | (wearable* or device* or sensor* or smart* or digital or mobile or acceleromet* or gyroscope* or (inertial adj2 measur* adj2 unit*) or "IMU" or magnetomet* or "compass" or "gps" or touchscreen* or (touch adj2 screen*) or camera* or "kinect" or "infrared").ti,ab,kw. | 102267 |
| 2 | (motor adj2 (skill* or function* or symptom* or sign* or impair* or control* or fluctuat*)).ti,ab,kw. | 12554 |
| 3 | 1 and 2 | 2591 |
| 4 | (book or chapter or editorial or erratum or letter or note or short survey or tombstone or comment or practice-guideline or journal correspondence or posters or news or newspaper article or lectures or interview or historical article or handbooks or guidelines or guidebooks or essays or database or catalogs).pt. | 9601 |
| 5 | 3 not 4 | 2590 |
| 6 | limit 5 to embase | 868 |
| 7 | limit 5 to medline | 1186 |
| 8 | 5 not (6 or 7) | 536 |
| 9 | limit 8 to english | 92 |
| 10 | limit 9 to yr="2015-current" | 85 |
| 11 | remove duplicates from 10 | 85 |

Table S5: Search strategy for US clinical trial registry (clinicaltrials.gov).

| **Database: clinicaltrials.gov**  **Search executed: October 2, 2020** | | |
| --- | --- | --- |
| **#** | **String** | **Hits** |
| 1 | **Other Terms:** “motor function”  **Study Results:** Studies With Results  **Age Group:** Adult (18-64); Older Adults (65+)  **Results First Posted:** From 01/01/2015 | 229 |

Table S6: Search strategy for EU clinical trial registry (clinicaltrialsregister.eu).

| **Database: clinicaltrialsregister.eu**  **Search executed: October 2, 2020** | | |
| --- | --- | --- |
| **#** | **String** | **Hits** |
| 1 | **Search Terms:** motor function  **Select Age Range:** Adult; Elderly  **Select Date Range:** 2015-01-01  **Results Status:** Trials with results | 27 |

Table S7: List of records excluded after full-text screening and the reason for exclusion.

| **First Author & Year** | **Title** | **Reason for Exclusion** |
| --- | --- | --- |
| Abram 2019 | Energy optimization is a major objective in the real-time control of step width in human walking | Intervention |
| Adams 2017 | Multiple wearable sensors in Parkinson and Huntington disease individuals: A pilot study in clinic and at home | Intervention |
| Adams 2018 | Evaluating wearable sensors for objective measurement of motor features of Huntington disease | Intervention |
| Adams 2018 | Virtual activities of daily living for recovery of upper extremity motor function | Intervention |
| Adams 2019 | Wearable sensors for the quantification of motor symptom in Parkinson disease and Huntington disease | Outcomes |
| Adams 2019 | Feasibility of using a smartphone application to evaluate Huntington disease | Outcomes |
| Adomaviciene 2019 | Influence of new technologies on post-stroke rehabilitation: A comparison of Armeo Spring to the Kinect system | Other |
| Adomaviciene 2019 | Kinect-based system in occupational therapy for hand motor functional recovery after stroke | Intervention |
| Aghanavesi 2018 | Objective assessment of Parkinson’s disease motor symptoms during leg agility test using motion sensors | Duplicate |
| Agostini 2015 | Does texting while walking really affect gait in young adults? | Intervention |
| Ahmad 2019 | Virtual reality games as an adjunct in improving upper limb function and general health among stroke survivors | Intervention |
| Aktar 2018 | Is there a relationship between depression/anxiety and physical inactivity in Parkinson disease? | Outcomes |
| Alfano 2018 | More than just fun and games: Active workspace volume video game quantifies upper extremity function in individuals with neuromuscular disease | Outcomes |
| Alonso De Lecinana 2018 | Evaluation of motor function impairment after stroke with a computational analysis system | Outcomes |
| Alt Murphy 2018 | Wearable sensors for clinical applications in stroke, Parkinson’s disease and epilepsy: A mixed-methods systematic review | Other |
| Althoff 2018 | Cognitive-motor interference in multiple sclerosis and its relation to cognitive and motor impairments | Outcomes |
| Alvarez-Lopez 2019 | Use of commercial off-the-shelf devices for the detection of manual gestures in surgery: Systematic literature review | Other |
| Amini 2019 | Kinect4fog: Monitoring and improving mobility in people with Parkinson’s using a novel system incorporating the Microsoft Kinect v2 | Intervention |
| Anand 2018 | Automatic detection of on/off states in Parkinson disease patients using wearable inertial sensors | Intervention |
| Andringa 2020 | Time course of wrist hyper-resistance in relation to upper limb motor recovery early post stroke | Intervention |
| Angeles 2017 | Automated assessment of symptom severity changes during deep brain stimulation (DBS) therapy for Parkinson’s disease | Intervention |
| Antonini 2018 | Acceptability to patients, carers and clinicians of an mHealth platform for the management of Parkinson’s disease (PD-manager): Study protocol for a pilot randomised controlled trial 11 medical and health sciences 1117 public health and health services | Study design |
| Aoki 2016 | Handedness and index finger movements performed on a small touchscreen | Intervention |
| Asakawa 2019 | Can the latest computerized technologies revolutionize conventional assessment tools and therapies for a neurological disease? The example of Parkinson's disease | Other |
| Austin and Siddall 2019 | Virtual reality for the treatment of neuropathic pain in people with spinal cord injuries: A scoping review | Other |
| Badawy 2019 | Metadata concepts for advancing the use of digital health technologies in clinical research | Study design |
| Bailey 2015 | Real-world affected upper limb activity in chronic stroke: An examination of potential modifying factors | Outcomes |
| Banca 2019 | A mobile phone app for the generation and characterization of motor habits | Outcomes |
| Bang 2019 | Inertial sensor-based tremor and bradykinesia quantification and potential for early disease identification in fragile x-associated tremor/ataxia syndrome (FXTAS) | Outcomes |
| Batista 2015 | Primary motor cortex representation of handgrip muscles in patients with leprosy | Intervention |
| Beck 2018 | Sparc: A new approach to quantifying gait smoothness in patients with Parkinson's disease | Outcomes |
| Beheshti 2019 | Unveiling' the effect of art therapy on eye movement phenotype in Parkinson's disease | Outcomes |
| Belvederi Murri 2020 | Instrumental assessment of balance and gait in depression: A systematic review | Other |
| Bergquist 2018 | West Sweden Parkinson objective measurement registry study (WESTPORTS) | Outcomes |
| Bhatnagar 2018 | Correlating home-based upper extremity activity monitoring with clinical evaluations for chronic moderate to severe hemiparesis post-stroke | Outcomes |
| Bhatnagar 2020 | Comparing home upper extremity activity with clinical evaluations of arm function in chronic stroke | Outcomes |
| Biagioni 2019 | Ambulatory inertial sensors in Parkinson's disease: Exploring the objective characterization of motor disability with timed up and go test | Intervention |
| Blumrosen 2016 | A real-time Kinect signature-based patient home monitoring system | Study design |
| Bobic 2019 | An expert system for quantification of bradykinesia based on wearable inertial sensors | Intervention |
| Bogard 2018 | Does altering medication to avoid dyskinesia positively alters the breadth of the motor repertoire of patients with Parkinson's disease? A pilot study | Outcomes |
| Bonnechère 2016 | The use of commercial video games in rehabilitation: A systematic review | Other |
| Bonnechère 2017 | Suitability of functional evaluation embedded in serious game rehabilitation exercises to assess motor development across lifespan | Population |
| Boot 2020 | Age-related Parkinsonian signs in microdeletion 22q11.2 | Intervention |
| Borbely and Szolgay 2017 | Real-time inverse kinematics for the upper limb: A model-based algorithm using segment orientations | Intervention |
| Boroojerdi 2019 | Clinical feasibility of a wearable, conformable sensor patch to monitor motor symptoms in Parkinson's disease | Intervention |
| Borot 2018 | Different hemodynamic responses of the primary motor cortex accompanying eccentric and concentric movements: A functional NIRS study | Intervention |
| Borschmann 2018 | Upright activity and higher motor function may preserve bone mineral density within 6 months of stroke: A longitudinal study | Outcomes |
| Botros 2019 | Long-term home-monitoring sensor technology in patients with Parkinson's disease-acceptance and adherence | Intervention |
| Bremer and Belmont 2018 | The emerging role of virtual reality platforms in physiotherapy rehabilitation for hemophilia patients | Outcomes |
| Brogioli 2016 | Monitoring upper limb recovery after cervical spinal cord injury: Insights beyond assessment scores | Outcomes |
| Buckley 2019 | The role of movement analysis in diagnosing and monitoring neurodegenerative conditions: Insights from gait and postural control | Other |
| Burka 2018 | A portable wireless motion capture system for patients with myotonic dystrophy | Intervention |
| Bykov and Bender 2019 | Bos-technologies of rehabilitation in patients with Parkinsonism | Outcomes |
| Camara 2018 | Eye movements in interception with delayed visual feedback | Intervention |
| Cao 2017 | Postural tremor and chronic inflammatory demyelinating polyneuropathy | Intervention |
| Capelini 2017 | Improvements in motor tasks through the use of smartphone technology for individuals with Duchenne muscular dystrophy | Population |
| do Carmo Vilas-Boas 2019 | TTR-FAP progression evaluation based on gait analysis using a single RGB-D camera | Study design |
| Carvalho 2017 | Robotic gait training for individuals with cerebral palsy: A systematic review and meta-analysis | Other |
| Cattaneo and Barchiesi 2015 | The auditory space in the motor system | Outcomes |
| Cerff 2017 | Home-based physical behavior in late stage Parkinson disease dementia: Differences between cognitive subtypes | Outcomes |
| Chen 2017 | Does task-oriented virtual reality training on chronic stroke patients decrease the resources utilization of physical therapy in Taiwan? | Outcomes |
| Chhabria 2018 | Amantadine extended release (GOCOVRI) reduces dyskinesia and bradykinesia: Evaluation of clinical response with the wearable PKG (personal Kinetigraph) watch | Outcomes |
| Chhabria and Isaacson 2018 | Clinical effect of the pkg watch in the management of Parkinson's patients | Outcomes |
| Chin 2020 | Upper limb use differs among people with varied upper limb impairment levels early post-stroke: A single-site, cross-sectional, observational study | Outcomes |
| Cho 2020 | Detecting motor function abnormalities in individuals with autism spectrum disorder without intellectual impairment via visual-perceptive computing | Outcomes |
| Choi 2020 | Effects of mechanical assistance on muscle activity and motor performance during isometric elbow flexion | Intervention |
| Chomiak 2017 | A training approach to improve stepping automaticity while dual-tasking in Parkinson's disease: A prospective pilot study | Intervention |
| Claridge 2015 | Quantification of physical activity and sedentary time in adults with cerebral palsy | Intervention |
| Cohen 2016 | Enabling breakthroughs in Parkinson's disease with wearable technologies and big data analytics | Other |
| Collado-Mateo 2016 | Performance of women with fibromyalgia in walking up stairs while carrying a load | Outcomes |
| Colli-Alfaro 2019 | Design of user-independent hand gesture recognition using multilayer perceptron networks and sensor fusion techniques | Intervention |
| Corra 2019 | Quantitative straight and circular walking parameters for detecting on and off medication states in early PD | Intervention |
| Coyle 2019 | The use of wearable devices to monitor motor disability in multiple sclerosis-a real-world pilot study | Intervention |
| Cucca 2019 | Can art therapy improve signs and symptoms of Parkinson's disease? Preliminary results from the EXPLORARTPD study | Outcomes |
| Dai 2015 | Quantitative assessment of Parkinsonian bradykinesia based on an inertial measurement unit | Intervention |
| Dai 2015 | Quantitative assessment of Parkinsonian tremor based on an inertial measurement unit | Intervention |
| Daneault 2018 | The levodopa response trial and the Parkinson disease digital biomarker challenge: Monitoring symptoms of Parkinson's disease in the lab and home using wearable sensors | Outcomes |
| Daneault 2018 | Could wearable and mobile technology improve the management of essential tremor? | Other |
| Danial-Saad 2019 | Hand function in skills of modern day among elderly individuals | Intervention |
| Dasgupta 2018 | You can tell by the way i use my walk. Predicting the presence of cognitive load with gait measurements | Outcomes |
| Dawe 2018 | Association between quantitative gait and balance measures and total daily physical activity in community-dwelling older adults | Outcomes |
| de Araujo 2019 | Efficacy of virtual reality rehabilitation after spinal cord injury: A systematic review | Other |
| de Freitas 2019 | Analysis of different device interactions in a virtual reality task in individuals with Duchenne muscular dystrophy-a randomized controlled trial | Outcomes |
| de Moraes 2020 | Motor learning and transfer between real and virtual environments in young people with autism spectrum disorder: A prospective randomized cross over controlled trial | Outcomes |
| Dechenaud 2019 | Development of adapted guitar to improve motor function after stroke: Feasibility study in young adults | Intervention |
| Deemer 2019 | Feasibility of testing visual motor function through a smart phone app in a comparative study evaluating head-mounted display systems | Outcomes |
| Del Din 2016 | Free-living monitoring of Parkinson's disease: Lessons from the field | Other |
| Delatorre and Marques 2019 | Mems-based measurement and classification system of tremors in Parkinson disease using MDS-UPDRS scale as a parameter | Intervention |
| Demanuele 2018 | Developing a self-administered instrumented motor exam for home-based Parkinson's disease assessment using wearable sensors | Outcomes |
| Demers and Levin 2018 | Reaching kinematics and affordances in a 2D virtual environment in post-stroke patients | Intervention |
| di Biase 2018 | Quantitative analysis of bradykinesia and rigidity in Parkinson's disease | Intervention |
| Di Lazzaro 2019 | Technology-based assessment of motor impairment in de novo Parkinson's disease patients: Implications for diagnostic accuracy and early identification of distinct phenotypes | Intervention |
| Di Lazzaro 2020 | Technology-based objective measures detect subclinical axial signs in untreated, de novo Parkinson's disease | Intervention |
| Dinesh 2020 | A longitudinal wearable sensor study in Huntington's disease | Intervention |
| Djuric-Jovicic 2018 | Finger and foot tapping sensor system for objective motor assessment | Intervention |
| Dockx 2016 | Virtual reality for rehabilitation in Parkinson's disease | Other |
| Dogan 2019 | Functional range of motion in the upper extremity and trunk joints: Nine functional everyday tasks with inertial sensors | Intervention |
| Doherty 2017 | Concussion is associated with altered preparatory postural adjustments during gait initiation | Intervention |
| Dominey and Carroll 2018 | Using remotely collected data to identify Parkinson's disease (PD) subtypes | Intervention |
| Donath 2016 | Effects of virtual reality training (EXERGAMING) compared to alternative exercise training and passive control on standing balance and functional mobility in healthy community-dwelling seniors: A meta-analytical review | Other |
| Ehsani 2019 | The association between cognition and dual-tasking among older adults: The effect of motor function type and cognition task difficulty | Intervention |
| Ekker 2016 | Neurorehabilitation for Parkinson's disease: Future perspectives for behavioural adaptation | Other |
| Eleftheriou 2018 | Circadian rhythm in idiopathic normal pressure hydrocephalus | Intervention |
| Erb 2018 | The BLUESKY project: Monitoring motor and non-motor characteristics of people with Parkinson's disease in the laboratory, a simulated apartment, and home and community settings | Intervention |
| Eskofier 2016 | Recent machine learning advancements in sensor-based mobility analysis: Deep learning for Parkinson's disease assessment | Outcomes |
| Fang 2019 | A novel multistandard compliant hand function assessment method using an infrared imaging device | Intervention |
| Felix 2020 | Objective sensor-based gait measures reflect motor impairment in multiple sclerosis patients: Reliability and clinical validation of a wearable sensor device | Duplicate |
| Ferraris 2019 | Feasibility of home-based automated assessment of postural instability and lower limb impairments in Parkinson's disease | Intervention |
| Ferreira 2017 | The effect of peripheral neuropathy on lower limb muscle strength in diabetic individuals | Intervention |
| Fino 2016 | A preliminary study of longitudinal differences in local dynamic stability between recently concussed and healthy athletes during single and dual-task gait | Study design |
| Fino 2018 | Abnormal turning and its association with self-reported symptoms in chronic mild traumatic brain injury | Intervention |
| Fischer and van den Heever 2016 | Portable video-oculography device for implementation in sideline concussion assessments: A prototype | Intervention |
| Fisher 2016 | Unsupervised home monitoring of Parkinson's disease motor symptoms using body-worn accelerometers | Intervention |
| FitzGerald 2018 | Quantifying motor impairment in movement disorders | Other |
| Flachenecker 2019 | Objective sensor-based gait measures reflect motor impairment in multiple sclerosis patients: Reliability and clinical validation of a wearable sensor device | Intervention |
| Flisar 2018 | Accelerometric evaluation of motor performance in pd patients before and after STN-DBS treatment | Intervention |
| Galperin 2019 | Associations between daily-living physical activity and laboratory-based assessments of motor severity in patients with falls and Parkinson's disease | Intervention |
| Garcia-Agundez 2019 | Recent advances in rehabilitation for Parkinson's disease with exergames: A systematic review | Other |
| Gatsios 2020 | Feasibility and utility of mHealth for the remote monitoring of Parkinson disease: Randomized controlled trial | Intervention |
| Gaul 2018 | It ain't what you do, it's the way that you do it: Does obesity affect perceptual motor control ability of adults on the speed and accuracy of a discrete aiming task? | Intervention |
| Ghazi 2018 | Surgeons eyes don't lie, utilizing eye tracking as a means to assess cognitive load during surgical simulation training | Outcomes |
| Ghoraani 2020 | Multilevel features for sensor-based assessment of motor fluctuation in Parkinson's disease subjects | Intervention |
| Gill 2016 | Changes in spatiotemporal gait patterns during flat ground walking and obstacle crossing 1 year after bariatric surgery | Intervention |
| Gizzi 2019 | People with low back pain show reduced movement complexity during their most active daily tasks | Intervention |
| Godinho 2016 | A systematic review of the characteristics and validity of monitoring technologies to assess Parkinson's disease | Other |
| Gohlke 2020 | Are changes in upper extremity use during sub-acute rehabilitation after stroke associated with physical, cognitive, and social activities? An observational cohort pilot study | Intervention |
| Gondo 2019 | Music therapy on gait disturbance and gait analysis for Parkinson's disease using a portable gait rhythmogram | Outcomes |
| Gooding 2018 | Digital, high-frequency, long-term monitoring of motor and nonmotor symptoms in Huntington disease patients | Outcomes |
| Goubault 2018 | What is the relationship between dyskinesia and performance of activities of daily living in patients with Parkinson's disease? | Intervention |
| Goubault 2018 | Cardinal motor features of Parkinson's disease coexist with peak-dose choreic-type drug-induced dyskinesia | Intervention |
| Grewal 2015 | Sensor-based interactive balance training with visual joint movement feedback for improving postural stability in diabetics with peripheral neuropathy: A randomized controlled trial | Intervention |
| Gulle 2019 | Muscle tibialis anterior fatigue protocol effects on kinetic and kinematic parameters of gait and balance: A laboratory study | Outcomes |
| Gutierrez-Zuniga 2018 | Computational analysis of movement for evaluation of motor function impairment after stroke | Outcomes |
| Haberfehlner 2020 | Instrumented assessment of motor function in dyskinetic cerebral palsy: A systematic review | Other |
| Haji Ghassemi 2019 | Turning analysis during standardized test using on-shoe wearable sensors in Parkinson's disease | Intervention |
| Hamacher 2016 | The reliability of local dynamic stability in walking while texting and performing an arithmetical problem | Intervention |
| Hannink 2017 | Benchmarking foot trajectory estimation methods for mobile gait analysis | Intervention |
| Hannink 2018 | Mobile stride length estimation with deep convolutional neural networks | Intervention |
| Harmsen 2015 | A mirror therapy-based action observation protocol to improve motor learning after stroke | Intervention |
| Hassani 2017? | Advanced 3d movement analysis algorithms for robust functional capacity assessment | Intervention |
| Hauser and Wade 2018 | Detecting reach to grasp activities using motion and muscle activation data | Intervention |
| Hee-Tae 2017 | Feasibility of using the Rapael smart glove in upper limb physical therapy for patients after stroke: A randomized controlled trial | Intervention |
| Heilbronn 2019 | Anticipatory postural adjustments are modulated by substantia nigra stimulation in people with Parkinson's disease and freezing of gait | Outcomes |
| Held 2018 | Inertial sensor measurements of upper-limb kinematics in stroke patients in clinic and home environment | Intervention |
| Heldberg 2015 | Using wearable sensors for semiology-independent seizure detection - towards ambulatory monitoring of epilepsy | Outcomes |
| Heldman 2016 | Computer-guided deep brain stimulation programming for Parkinson's disease | Outcomes |
| Heldman 2017 | Telehealth management of Parkinson's disease using wearable sensors: An exploratory study | Intervention |
| Hemmati and Wade 2016 | Detecting postural transitions: A robust wavelet-based approach | Intervention |
| Henchoz 2015 | Energetics and mechanics of walking in patients with chronic low back pain and healthy matched controls | Intervention |
| Herold 2017 | Cortical activation during balancing on a balance board | Intervention |
| Hesam-Shariati 2019 | Improved kinematics and motor control in a longitudinal study of a complex therapy movement in chronic stroke | Intervention |
| Honda 2019 | Objective assessment and rating of cerebellar ataxia measured by a depth sensor | Intervention |
| Honda 2020 | Assessment and rating of motor cerebellar ataxias with the Kinect v2 depth sensor: Extending our appraisal | Study design |
| Hssayeni 2019 | Activity-independent detection of mediation states in individuals with Parkinson's disease using wearable sensors | Outcomes |
| Hu and Chomiak 2019 | Wearable technological platform for multidomain diagnostic and exercise interventions in Parkinson's disease | Other |
| Huang 2018 | Use of whole body vibration in individuals with chronic stroke: Transmissibility and signal purity | Intervention |
| Hubble 2016 | Assessing stability in mild and moderate Parkinson's disease: Can clinical measures provide insight? | Intervention |
| Huisinga 2018? | An instrumented timed up and go in facioscapulohumeral muscular dystrophy | Intervention |
| Hulbert 2019 | Digital dancing'-can you see what you feel?: An exploration of the physical 'experience' of dance for Parkinson's through 3-d motion analysis | Outcomes |
| Hung 2019 | Comparison of Kinect2scratch game-based training and therapist-based training for the improvement of upper extremity functions of patients with chronic stroke: A randomized controlled single-blinded trial | Intervention |
| Hussain 2018 | Upper limb kinematics in stroke and healthy controls using target-to-target task in virtual reality | Outcomes |
| Iakovakis 2019 | Towards unobtrusive Parkinson's disease detection via motor symptoms severity inference from multimodal smartphonesensor data | Outcomes |
| Ilias 2017 | Using measurements from wearable sensors for automatic scoring of Parkinson's disease motor states: Results from 7 patients | Study design |
| Isaacson 2019 | Effect of using a wearable device on clinical decision-making and motor symptoms in patients with Parkinson's disease starting transdermal rotigotine patch: A pilot study | Intervention |
| Iwaki 2019 | Using spontaneous eye-blink rates to predict the motor status of patients with Parkinson's disease | Study design |
| Jensen 2018 | Mobile sensor-based gait analysis provides objective motor assessments in Huntington's disease | Outcomes |
| Jinuk 2017 | Quantitative assessment test for upper-limb motor function by using EMG and kinematic analysis in the practice of occupational therapy | Outcomes |
| Johansson 2018 | Wearable sensors for clinical applications in epilepsy, Parkinson's disease, and stroke: A mixed-methods systematic review | Other |
| Johansson 2019 | Evaluation of device-assisted treatment using a wearable accelerometry wrist sensor | Outcomes |
| Johansson 2019 | Evaluation of a sensor algorithm for motor state rating in Parkinson's disease | Intervention |
| Johnston 2017 | Inertial sensor technology can capture changes in dynamic balance control during the y balance test | Intervention |
| Johnston 2019 | Investigating normal day to day variations of postural control in a healthy young population using Wii balance boards | Outcomes |
| Joshi 2019 | Pkg movement recording system use shows promise in routine clinical care of patients with Parkinson's disease | Other |
| Junghans and Khuu 2019 | Populations norms for "slurp"-an iPad app for quantification of visuomotor coordination testing | Intervention |
| Kakei 2018 | Quantitative evaluation of motor function and its clinical application | Intervention |
| Kalron 2015 | Validity and test-retest reliability of a measure of hand sensibility and manual dexterity in people with multiple sclerosis: The resense test | Intervention |
| Kelleran 2016 | Three-dimensional assessment of postural tremor during goal-directed aiming | Outcomes |
| Khoshnam 2018 | Effects of galvanic vestibular stimulation on upper and lower extremities motor symptoms in Parkinson's disease | Intervention |
| Kim 2019 | A comparison of activity monitor data from devices worn on the wrist and the waist in people with Parkinson's disease | Intervention |
| Kizony 2017 | Tele-rehabilitation service delivery journey from prototype to robust in-home use | Outcomes |
| Konig 2017 | Objective measurement of gait parameters in healthy and cognitively impaired elderly using the dual-task paradigm | Outcomes |
| Kostikis 2020 | PDmonitor: A novel system for objective monitoring of Parkinson's disease symptoms-efficacy and usability study | Intervention |
| Kramer 2018 | Long-term ambulatory assessment of motor symptoms in movement disorders: A best-evidence review | Other |
| Kumar 2016 | Smarteye: Developing a novel eye tracking system for quantitative assessment of oculomotor abnormalities | Intervention |
| Labaran 2019 | 87. Effects of spinal decompression on the gait efficiency and balance of cervical spondylotic myelopathy patients: Preliminary results | Intervention |
| Lakhani 2017 | Hemispheric asymmetry in myelin after stroke is related to motor impairment and function | Outcomes |
| Lam 2019 | Smartphone-based assessment of fatigue and fatigability in multiple sclerosis | Outcomes |
| Lancaster 2019 | Smartphone-based assessment of executive function: Analysing motor-response trajectories to enhance the detection of preclinical cognitive impairment | Intervention |
| Lancioni 2019 | Recent technology-aided programs to support adaptive responses, functional activities, and leisure and communication in people with significant disabilities | Other |
| Lancioni 2020 | A tablet-based program to enable people with intellectual and other disabilities to access leisure activities and video calls | Intervention |
| Lavelle 2020 | Validity of the international physical activity questionnaire short form (ipaq-sf) as a measure of physical activity (pa) in young people with cerebral palsy: A cross-sectional study | Population |
| Laver 2017 | Virtual reality for stroke rehabilitation | Other |
| Leal 2020 | The use of a task through virtual reality in cerebral palsy using two different interaction devices (concrete and abstract) - a cross-sectional randomized study | Population |
| Lebel 2019 | Parkinson's disease patients experiencing peak-dose dyskinesia redistribute involuntary movements throughout their body to improve motor control | Intervention |
| Lee 2017 | Subjective perception of sleep benefit in Parkinson's disease: Valid or irrelevant? | Outcomes |
| Lee 2017 | Objective measurement and characterization of sleep benefit in Parkinson's disease | Intervention |
| Lee 2018 | Automated evaluation of upper-limb motor function impairment using fugl-meyer assessment | Intervention |
| Lee 2018 | Towards the ambulatory assessment of movement quality in stroke survivors using a wrist-worn inertial sensor | Intervention |
| Lee 2020 | Gait characteristics under imposed challenge speed conditions in patients with Parkinson's disease during overground walking | Intervention |
| Li 2016 | A single task assessment system of upper-limb motor function after stroke | Intervention |
| Li 2017 | Motor function evaluation of hemiplegic upper-extremities using data fusion from wearable inertial and surface EMG sensors | Intervention |
| Li 2019 | Use digital sensor and deep learning to evaluate motor performance in the d1pam (ly3154207) phase 1b Parkinson's disease clinical trial | Outcomes |
| Linder 2019 | A mobile device dual-task paradigm for the assessment of MTBI | Intervention |
| Lipsmeier 2018 | Digital, high-frequency, long-term monitoring of motor and non-motor symptoms in Huntington's disease (HD) patients | Outcomes |
| Lipsmeier 2018 | Remote patient monitoring with a digital biomarker approach generates clinically distinctive and meaningful sensor feature data in Parkinson's disease: Differential relationships with MDS-UPDRS-III, PDQ-39 and DAT-SPECT | Outcomes |
| Lipsmeier 2018 | Remote patient testing with smartphones provides reliable, valid and sensitive measures of motor symptom severity in Parkinson's disease patients | Outcomes |
| Lipsmeier 2019 | Reliability, feasibility and validity of a novel digital monitoring platform assessing cognitive and motor symptoms in people with stage I and II Huntington's disease (HD) | Outcomes |
| Lipsmeier 2019 | Preliminary validation of a novel, comprehensive digital biomarker smartphone application to assess motor symptoms in de-novo diagnosed Parkinson patients | Duplicate |
| Lipsmeier 2020 | Passively measuring motor behavior in daily life: Preliminary reliability and validity in individuals recently diagnosed with Parkinson's disease | Outcomes |
| Liu 2019 | Design of virtual guiding tasks with haptic feedback for assessing the wrist motor function of patients with upper motor neuron lesions | Intervention |
| Liu 2019 | Vision-based method for automatic quantification of Parkinsonian bradykinesia | Intervention |
| Lo 2018 | The use of smartphone task derived features to predict clinical scores in Parkinson's disease (pd) | Duplicate |
| Loprinzi 2018 | A pilot study evaluating the association between physical activity and cognition among individuals with Parkinson's disease | Other |
| Lynch 2019 | Continuous objective monitoring in Parkinson's disease: A description of over 25,000 Parkinson's symptom scores across the world using the personal kinetigraph (PKG) wearable monitoring device | Outcomes |
| Ma 2019 | SEMG-based trunk compensation detection in rehabilitation training | Intervention |
| Maceira-Elvira 2019 | Wearable technology in stroke rehabilitation: Towards improved diagnosis and treatment of upper-limb motor impairment | Other |
| MacRitchie and McPherson 2015 | Integrating optical finger motion tracking with surface touch events | Intervention |
| Madrid-Navarro 2018 | Multidimensional circadian monitoring by wearable biosensors in Parkinson's disease | Intervention |
| Maetzler 2019 | Wearables for monitoring pd and its treatment | Other |
| Maij 2017 | Afferent motor feedback determines the perceived location of tactile stimuli in the external space presented to the moving arm | Intervention |
| Maldonado-Naranjo 2018 | Quantitative kinematic analysis of tremor and bradykinesia in patients with Parkinson disease using a wirelessstylus | Intervention |
| Malhotra 2020 | Is the Parkinson's kinetigraph reflective of clinical off/on motor testing: Single center experience | Intervention |
| Mantri 2018 | Motivators, barriers and clinical correlates of physical activity in Parkinson disease | Outcomes |
| Martinikorena 2016 | Gait variability related to muscle quality and muscle power output in frail nonagenarian older adults | Intervention |
| Massetti 2018 | The clinical utility of virtual reality in neurorehabilitation: A systematic review | Other |
| Matias 2017 | A perspective on wearable sensor measurements and data science for Parkinson's disease | Other |
| Matic and Gomez-Marin 2019 | A customizable tablet app for hand movement research outside the lab | Other |
| McPhee 2017 | Fatigue and its relationship with physical activity, age, and body composition in adults with cerebral palsy | Intervention |
| Mehrang 2018 | Identification of Parkinson's disease utilizing a single self-recorded 20-step walking test acquired by smartphone's inertial measurement unit | Outcomes |
| Melendez-Calderon 2017 | Transfer of dynamic motor skills acquired during isometric training to free motion | Intervention |
| Melo 2019 | Hand motor slowness in Parkinson disease patients performing fitts task | Intervention |
| Memedi 2015 | Automatic spiral analysis for objective assessment of motor symptoms in Parkinson's disease | Intervention |
| Menozzi 2019 | Smartphone software for home monitoring of motor symptoms in Parkinson's disease-the cloudUPDRS smartphone software in Parkinson's (CUSSP) study | Intervention |
| Miller Koop 2019 | Mobility improves after high intensity aerobic exercise in individuals with Parkinson's disease | Intervention |
| Millor 2020 | High density muscle size and muscle power are associated with both gait and sit-to-stand kinematic parameters in frail nonagenarians | Outcomes |
| Mirelman 2019 | Objective quantifiable assessment of nocturnal movements in patients with Parkinson's disease using a wearable sensor | Intervention |
| Mitsutake 2020 | Standing postural stability during galvanic vestibular stimulation is associated with the motor function of the hemiplegic lower extremity post-stroke | Intervention |
| Miyawaki 2020 | Agency judgments in post-stroke patients with sensorimotor deficits | Intervention |
| Mohanty 2019 | Mindfulness training to reduce anxiety in Parkinson's disease: A cautionary tale about wearable technology | Outcomes |
| Morgan 2020 | Systematic review looking at the use of technology to measure free-living symptom and activity outcomes in Parkinson's disease in the home or a home-like environment | Other |
| Morgante 2019 | Shedding light on the relationship between dyskinesia assessed by a wearable device and impulsive compulsive behaviour in Parkinson's disease | Outcomes |
| Morris 2019 | The interplay between cholinergic activity, attention, and turning in Parkinson's disease | Outcomes |
| Morrison 2015 | Usability and acceptability of assess MS: Assessment of motor dysfunction in multiple sclerosis using depth-sensing computer vision | Intervention |
| Mrabet 2019 | Oculomotor function in amyotrophic lateral sclerosis | Intervention |
| Murali 2018 | Wireless recording of limb flexion-extension counter using gsm module | Intervention |
| Nackaerts 2020 | Retention of touchscreen skills is compromised in Parkinson's disease | Intervention |
| Naghavi 2019 | Towards real-time prediction of freezing of gait in patients with Parkinson's disease: Addressing the class imbalance problem | Intervention |
| Narai 2016 | Accelerometer-based monitoring of upper limb movement in older adults with acute and subacute stroke | Intervention |
| Nelson and Wade 2018 | Relative efficacy of sensor modalities for estimating post-stroke motor impairment | Outcomes |
| Nene 2019 | Ambulatory monitoring of electrodermal activity (EDA) and heart rate variability (HRV) for prediction of motor fluctuations in Parkinson's disease (PD) | Intervention |
| Nero 2016 | Objectively assessed physical activity and its association with balance, physical function and dyskinesia in Parkinson's disease | Intervention |
| Nguyen 2019 | Development and clinical validation of inertial sensor-based gait-clustering methods in Parkinson's disease | Intervention |
| Niechwiej-Szwedo 2018 | Evaluation of the leap motion controller during the performance of visually-guided upper limb movements | Intervention |
| Nishikawa 2018 | Neuromodulation therapy for the treatment of gait disturbance | Intervention |
| Nuic 2018 | The feasibility and positive effects of a customised videogame rehabilitation programme for freezing of gait and falls in Parkinson's disease patients: A pilot study | Intervention |
| Oliveira 2019 | A software for testing and training visuo-motor coordination for upper limb control | Intervention |
| Ona 2018 | Automatic outcome in manual dexterity assessment using colour segmentation and nearest neighbour classifier | Intervention |
| Ono 2019 | Asymmetric smooth pursuit eye movements and visual motion reaction time | Intervention |
| Ono and Kizuka 2017 | Effects of visual error timing on smooth pursuit gain adaptation in humans | Intervention |
| Orand 2019 | Bilateral tactile feedback-enabled training for stroke survivors using Microsoft Kinect<sup>tm</sup> | Study design |
| Ordnung 2017 | No overt effects of a 6-week exergame training on sensorimotor and cognitive function in older adults. A preliminary investigation | Intervention |
| Ossig 2016 | Wearable sensor-based objective assessment of motor symptoms in Parkinson's disease | Other |
| Ossmy and Mukamel 2017 | Short term motor-skill acquisition improves with size of self-controlled virtual hands | Intervention |
| Otte 2018 | Instrumental assessment of upper extremity bradykinesia in UPDRS testing based on depth video data | Outcomes |
| Otte 2019 | Cultural bias in performance of motor tasks? Comparison between Japanese and German healthy adults | Intervention |
| Ozana 2018 | Grasping trajectories in a virtual environment adhere to weber's law | Intervention |
| Ozturk 2018 | Quantitative keyboard tapping measurement correlates with electrophysiological changes in STN LFPS of PD patients treated with L-DOPA better than UPDRS subscores | Intervention |
| Pahwa 2018 | Evaluating long-term effectiveness of carbidopa/levodopa enteral suspension in advanced Parkinson's disease patients: Provide study design and baseline characteristics | Intervention |
| Pahwa 2018 | Using objective measurement via wearable sensors to evaluate real-world effectiveness of carbidopa/levodopa enteral suspension for managing motor fluctuations: Provide study design and baseline characteristics | Outcomes |
| Pahwa 2019 | Evaluating the real-world impact of levodopa/carbidopa intestinal gel (LCIG) on motor symptoms using wearable sensors: Evidence from provide study | Intervention |
| Palmer 2015 | A randomised trial into the effect of an isolated hip abductor strengthening programme and a functional motor control programme on knee kinematics and hip muscle strength | Intervention |
| Papapetropoulos 2019 | A phase 2, randomized, double-blind, placebo-controlled trial of cx-8998, a selective modulator of the t-type calcium channel in inadequately treated moderate to severe essential tremor: T-calm study design and methodology for efficacy endpoint and digital biomarker selection | Study design |
| Picardi 2019 | Instrumental timed up and go test measures for falls risk assessment in stroke patients | Outcomes |
| Pilloni 2020 | Gait and functional mobility in multiple sclerosis: Immediate effects of transcranial direct current stimulation (TDCS) paired with aerobic exercise | Intervention |
| Pogrzeba 2019 | Analysis and quantification of repetitive motion in long-term rehabilitation | Intervention |
| Pop-Jordanova 2017 | The use of smartphone in medical practice | Other |
| Porta 2018 | Association between objectively measured physical activity and gait patterns in people with Parkinson's disease: Results from a 3-month monitoring | Outcomes |
| Pulliam 2015 | Motion sensor strategies for automated optimization of deep brain stimulation in Parkinson's disease | Outcomes |
| Pulliam 2018 | Continuous assessment of levodopa response in Parkinson's disease using wearable motion sensors | Intervention |
| Purcell 2020 | The effects of dual-task cognitive interference on gait and turning in Huntington's disease | Intervention |
| Quezada 2017 | Usability operations on touch mobile devices for users with autism | Outcomes |
| Rabelo 2017 | Objective assessment of bradykinesia estimated from the wrist extension in older adults and patients with Parkinson's disease | Other |
| Raju 2019 | Freezing of gait in Parkinsons disease: A deterministic model classification objective method for tracking and predicting preventing fog episodes in PDS (prognostic diagnosis) | Outcomes |
| Raju and Kandadai 2019 | Tracking freezing of gait in Parkinson's disease: A model identification objective method for predicting and preventing fog episodes in pd | Outcomes |
| Rama Raju and Borgohain 2018 | STN DBS - a study of frequency of gait (fog) in Parkinson's disease | Outcomes |
| Rand and Eng 2015 | Predicting daily use of the affected upper extremity 1 year after stroke | Intervention |
| Rech 2020 | Fugl-meyer assessment scores are related with kinematic measures in people with chronic hemiparesis after stroke | Intervention |
| Robertson 2020 | Tremorography in fragile x-associated tremor/ataxia syndrome, Parkinson's disease and essential tremor | Intervention |
| Rodríguez-Martín 2019 | Stat-on: A wearable inertial system to objectively evaluate motor symptoms in Parkinson's disease | Other |
| Rodríguez-Molinero 2018 | A kinematic sensor and algorithm to detect motor fluctuations in Parkinson disease: Validation study under real conditions of use | Intervention |
| Rodríguez-Molinero 2019 | Randomized multicenter single-blind parallel-group trial to compare the efficacy of a holter for Parkinson symptoms against other clinical follow-up methods | Outcomes |
| Rourke 2020 | How does virtual reality simulation compare to simulated practice in the acquisition of clinical psychomotor skills for pre-registration student nurses? A systematic review | Other |
| Rovini 2017 | How wearable sensors can support Parkinson's disease diagnosis and treatment: A systematic review | Other |
| Rovini 2018 | Comparative motor pre-clinical assessment in Parkinson's disease using supervised machine learning approaches | Intervention |
| Rovini 2019 | Fine motor assessment with unsupervised learning for personalized rehabilitation in Parkinson disease | Intervention |
| Rubin 2019 | Patient centered outcomes analysis for multiple sclerosis using a mobile application | Outcomes |
| Saether 2015 | The relationship between trunk control in sitting and during gait in children and adolescents with cerebral palsy | Population |
| Sarfo 2018 | Tele-rehabilitation after stroke: An updated systematic review of the literature | Other |
| Sato 2018 | Rapid foot-tapping but not hand-tapping ability distinguishes between multiple sclerosis subtypes | Duplicate |
| Sato 2020 | Rapid foot-tapping but not hand-tapping ability is different between relapsing-remitting and progressive multiple sclerosis | Intervention |
| Sayre 2018 | Assessing age-related differences in frailty measurements among pokot agro-pastoralists of Kenya | Outcomes |
| Scano 2017 | Kinect v2 performance assessment in daily-life gestures: Cohort study on healthy subjects for a reference database for automated instrumental evaluations on neurological patients | Intervention |
| Schwenk 2016 | Interactive sensor-based balance training in older cancer patients with chemotherapy-induced peripheral neuropathy: A randomized controlled trial | Intervention |
| Seok and Kwon 2019 | Extraterritorial motor function in carpal tunnel syndrome: A study with angular rate measurement system based on gyrosensor | Intervention |
| Sharma and Sharma 2019 | A smart device system (wearable technology band) to identify new phenotypical characteristics in Parkinson's disease patients | Intervention |
| Shawen 2020 | Role of data measurement characteristics in the accurate detection of Parkinson's disease symptoms using wearable sensors | Intervention |
| Shen 2019 | A pilot study to evaluate the severity of motor dysfunction in patients with Parkinson's disease based on ai non-wearable motion capture of video analysis | Intervention |
| Shi 2019 | Artificial intelligence in the monitoring and management of MS | Other |
| Shim and Jung 2015 | Effects of bilateral training on motor function, amount of activity and activity intensity measured with an accelerometer of patients with stroke | Intervention |
| Shires 2016 | Evaluating the Microsoft Kinect for use in upper extremity rehabilitation following stroke as a commercial off-the-shelf gaming system | Other |
| Silva Cunha 2016 | A novel portable, low-cost Kinect-based system for motion analysis in neurological diseases | Study design |
| Silva de Lima 2018 | Impact of motor fluctuations on real-life gait in Parkinson's patients | Intervention |
| Smith Fine 2019 | Wearable sensors detect impaired balance and coordination in lbsl during remote, home-based assessments | Intervention |
| Smulders 2019 | Effects of a physical rehabilitation program with cognitive challenge for freezing of gait - a pilot study | Outcomes |
| Snir 2018 | The ALS mobile analyzer: Monitor disease progression using a mobile app | Intervention |
| Snyder 2018 | A novel objective measure for measuring chorea in Huntington disease | Intervention |
| Souza 2020 | The use of the gait profile score and gait variable score in individuals with Duchenne muscular dystrophy | Intervention |
| Spasojevic 2017 | Quantitative assessment of the arm/hand movements in Parkinson's disease using a wireless armband device | Intervention |
| Spear 2018 | Feasibility of smartphone application use for the objective evaluation of the effects of Huntington's disease on motor function | Outcomes |
| Straaten 2018 | Reliability of 3D lower extremity movement analysis by means of inertial sensor technology during transitional tasks | Intervention |
| Summa 2017 | Assessing bradykinesia in Parkinson's disease using gyroscope signals | Intervention |
| Taylor 2018 | A comprehensive digital biomarker active testing and passive monitoring suite for the remote and frequent assessment of motor symptom progression in Parkinson's disease | Outcomes |
| Terashi 2019 | Relationship between daily physical activity measured by a triaxial accelerometer and motor symptoms in patients with Parkinson's disease | Outcomes |
| Teshuva 2019 | Using wearables to assess bradykinesia and rigidity in patients with Parkinson's disease: A focused, narrative review of the literature | Other |
| Thiede 2016 | Gait and balance assessments as early indicators of frailty in patients with known peripheral artery disease | Outcomes |
| Thomann 2020 | Passive monitored daily motor behavior significantly relates to quality of life in individuals with early Parkinson's disease | Outcomes |
| Tinius 2020 | Maternal factors related to infant motor development at 4 months of age | Population |
| Toosizadeh 2015 | Motor performance assessment in Parkinson's disease: Association between objective in-clinic, objective in-home, and subjective/semi-objective measures | Intervention |
| Toosizadeh 2016 | Alterations in gait parameters with peripheral artery disease: The importance of pre-frailty as a confounding variable | Intervention |
| Toosizadeh 2019 | Screening older adults for amnestic mild cognitive impairment and early-stage Alzheimer’s disease using upper-extremity dual-tasking | Intervention |
| Torres 2018 | Statistical platform for individualized behavioral analyses using biophysical micro-movement spikes | Other |
| Tortelli 2019 | The DIGITAL-HD study: Smartphone-based remote testing to assess cognitive and motor symptoms in Huntington's disease | Outcomes |
| Trevizan 2018 | Efficacy of different interaction devices using non-immersive virtual tasks in individuals with amyotrophic lateral sclerosis: A cross-sectional randomized trial | Intervention |
| Van Ancum 2019 | Gait speed assessed by a 4-m walk test is not representative of daily-life gait speed in community-dwelling adults | Intervention |
| Van Wamelen 2019 | Wearable sensor (Parkinson's kinetigraph) and dopamine transporter imaging as potential biosignature for constipation in Parkinson's | Intervention |
| Varghese 2019 | A smart device system to identify new phenotypical characteristics in movement disorders | Study design |
| Velasco 2018 | Gait and stance events measured with smartwatches in patients with pd and et | Intervention |
| Waddell 2018 | Feasibility of objectively evaluating Huntington disease's motor effects via smartphone application | Outcomes |
| Waddell 2019 | George-the first smartphone application for Huntington disease-pilot study | Outcomes |
| Wasse lius 2019 | Stroke detection by wearable accelerometers-proof of concept of a stroke alarm | Intervention |
| Ye 2018 | Quantitative assessment of the hand motor symptoms in Parkinson's disease based on a custom wearable device: A proof-of-principle study | Intervention |
| Zach 2018 | The occurrence of dopamine-responsive and dopamine-resistant resting tremor in Parkinson's disease | Outcomes |
| Zhou 2018 | Hemodialysis impact on motor function beyond aging and diabetes-objectively assessing gait and balance by wearable technology | Intervention |
| Zhou 2018 | Motor planning error: Toward measuring cognitive frailty in older adults using wearables | Intervention |
| Zhou 2019 | Motor function and physical frailty in hemodialysis patients with severe obesity | Outcomes |

Table S8: Key study characteristics of studies included after full-text screening.

| **First Author & Year** | **Title** | **Study Setting** | **N** | **Follow-up** |
| --- | --- | --- | --- | --- |
| Abrami 2020 [27] | Using an unbiased symbolic movement representation to characterize Parkinson’s disease states | Laboratory; Home | 80 | 6 days |
| Adams 2015 [28] | Assessing upper extremity motor function in practice of virtual activities of daily living | Laboratory | 14 | 2 weeks |
| Aghanavesi 2020a [80]; Aghanavesi 2020b [79] | Motion sensor-based assessment of Parkinson’s disease motor symptoms during leg agility tests: Results from levodopa challenge | Laboratory | 19 | Cross-sectional |
|  | A multiple motion sensors index for motor state quantification in Parkinson’s disease | Laboratory | 19 | Cross-sectional |
| Ahlrichs 2016 [93] | Detecting freezing of gait with a tri-axial accelerometer in Parkinson’s disease patients | Home | 20 | Cross-sectional |
| Alberts 2015 [29] | Using accelerometer and gyroscopic measures to quantify postural stability | Laboratory | 49 | Cross-sectional |
| Andrzejewski 2016 [99] | Wearable sensors in Huntington disease: A pilot study | Laboratory; Home | 20 | 7 days |
| Arora 2018 [42] | Smartphone motor testing to distinguish idiopathic rem sleep behavior disorder, controls, and pd | Laboratory; Home | 522 | 7 days |
| Arroyo-Gallego 2017 [103] | Detection of motor impairment in Parkinson’s disease via mobile touchscreen typing | Laboratory | 51 | Cross-sectional |
| Arroyo-Gallego 2018 [59] | Detecting motor impairment in early Parkinson’s disease via natural typing interaction with keyboards: Validation of the neuroQWERTY approach in an uncontrolled at-home setting | Laboratory; Home | 60 | 7 days |
| Beange 2019 [73] | Concurrent validity of a wearable IMU for objective assessments of functional movement quality and control of the lumbar spine | Laboratory | 10 | Cross-sectional |
| Bennasar 2018 [43] | Automated assessment of movement impairment in Huntington’s disease | Laboratory | 92 | Cross-sectional |
| Bernad-Elazari 2016 [87] | Objective characterization of daily living transitions in patients with Parkinson’s disease using a single body-fixed sensor | Laboratory; Home | 137 | 3 days (considered cross-sectional) |
| Bonnechère 2018 [113] | Automated functional upper limb evaluation of patients with Friedrich ataxia using serious games rehabilitation exercises | Laboratory | 27 | Cross-sectional |
| Boukhvalova 2018 [25] | Identifying and quantifying neurological disability via smartphone | Laboratory; Home | 31 | 9 weeks |
| Brooks 2020 [30] | Variations in rest-activity rhythm are associated with clinically measured disease severity in Parkinson’s disease | Laboratory; Home | 15 | 2 weeks |
| Campos 2018 [74] | Validity of the Actigraph activity monitor for individuals who walk slowly post-stroke | NR | 53 | <1 days (considered cross-sectional) |
| Capecci 2016 [54] | A smartphone-based architecture to detect and quantify freezing of gait in Parkinson’s disease | Laboratory | 20 | Cross-sectional |
| Cavallo 2019 [55] | Upper limb motor pre-clinical assessment in Parkinson’s disease using machine learning | Laboratory | 90 | Cross-sectional |
| Chae 2020 [63] | Development and clinical evaluation of web-based upper-limb home rehabilitation system using smartwatch and machine-learning model for chronic stroke survivors: Development, usability, and comparative study | Home | 38 | 18 weeks |
| Christiansen 2017 [31] | Factors associated with ambulatory activity in de novo Parkinson disease | NR | 128 | 10 days |
| Coates 2020 [44];  Pantall 2018a [50];  Pantall 2018b [51] | Entropy of real-world gait in Parkinson’s disease determined from wearable sensors as a digital marker of altered ambulatory behavior | Laboratory | 10 | 36 months |
|  | Longitudinal changes over thirty-six months in postural control dynamics and cognitive function in people with Parkinson’s disease | Laboratory | 109 | 54 months |
|  | Postural dynamics are associated with cognitive decline in Parkinson’s disease | Laboratory | 35 | 54 months |
| de Paula 2018 [84] | Motor performance of individuals with cerebral palsy in a virtual game using a mobile phone | NR | 50 | Cross-sectional |
| Dehbandi 2017 [32] | Using data from the Microsoft Kinect 2 to quantify upper limb behavior: A feasibility study | Laboratory | 24 | Cross-sectional |
| Del Din 2018a [45] | Free-living gait in rem sleep behaviour disorder: Measures of prodromal Parkinson’s disease? | NR | 98 | 7 days |
| Del Din 2018b [46] | Instrumented gait analysis identifies potential predictors for Parkinson’s disease converters | Laboratory | 64 | 8 years |
| Dowling 2018 [33] | Quantitative assessment of appendicular bradykinesia in Parkinson's disease using wearable sensors | Laboratory | 8 | Cross-sectional |
| Evers 2019 [75] | Real-life gait performance as a marker for motor fluctuations: The Parkinson@Home validation study | Home | 50 | Cross-sectional |
| Gordon 2019 [107] | Quantification of motor function in Huntington disease patients using wearable sensor devices | Laboratory; Home | 15 | 6 months |
| Hasan 2019 [47] | The bradykinesia akinesia incoordination (BRAIN) tap test: Capturing the sequence effect | NR | 194 | Cross-sectional |
| Hiorth 2016 [91] | Impact of falls on physical activity in people with Parkinson's disease | Home | 50 | 7 days |
| Horigome 2020 [70] | Evaluating the severity of depressive symptoms using upper body motion captured by RGB-depth sensors and machine learning in a clinical interview setting: A preliminary study | Laboratory | 47 | Cross-sectional |
| Horne 2015 [100] | An objective fluctuation score for Parkinson's disease | Laboratory | 565 | Cross-sectional |
| Hssayeni 2018 [114] | Deep learning for medication assessment of individuals with Parkinson's disease using wearable sensors | NR | 19 | Cross-sectional |
| Hssayeni 2019 [34] | Assessment of response to medication in individuals with Parkinson's disease | Laboratory | 19 | Cross-sectional |
| Hsu 2019 [81] | Gait and trunk movement characteristics of chronic ischemic stroke patients | Laboratory | 20 | Cross-sectional |
| Huang 2019 [82] | Validity of a novel touch screen tablet-based assessment for mild cognitive impairment and probable ad in older adults | NR | 120 | Cross-sectional |
| Hughes 2019 [35] | Quantitative assessment of upper limb motor function in Ethiopian acquired brain injured patients using a low-cost wearable sensor | NR | 14 | Cross-sectional |
| Iakovakis 2019 [89];  Iakovakis 2018 [88] | Early Parkinson's disease detection via touchscreen typing analysis using convolutional neural networks | Laboratory | 33 | Cross-sectional |
|  | Touchscreen typing-pattern analysis for detecting fine motor skills decline in early-stage Parkinson's disease | Laboratory | 33 | Cross-sectional |
| Kassavetis 2016 [28] | Developing a tool for remote digital assessment of Parkinson's disease | Laboratory | 14 | Cross-sectional |
| Kim 2015 [111] | Unconstrained detection of freezing of gait in Parkinson's disease patients using smartphone | NR | NR | Cross-sectional |
| Kim 2018 [64] | Validation of freezing-of-gait monitoring using smartphone | Laboratory | 32 | Cross-sectional |
| Klingelhoefer 2019 [94] | Medical evaluation as gold standard to control iPrognosis application derived data for early Parkinson's disease detection | Laboratory | 1465 | 6 months |
| Lalvay 2017 [95] | Quantitative measurement of akinesia in Parkinson's disease | Laboratory | 465 | Cross-sectional |
| Lauraitis 2020 [90] | A mobile application for smart computer-aided self-administered testing of cognition, speech, and motor impairment | Home | 15 | Cross-sectional |
| Lee 2016a [90] | A validation study of a smartphone-based finger tapping application for quantitative assessment of bradykinesia in Parkinson's disease | NR | 144 | Cross-sectional |
| Lee 2016b [83] | Validation of a smartphone application measuring motor function in Parkinson's disease | Laboratory; Home | 103 | 1-2 weeks |
| Lee 2018 [65] | Detection of hemiplegic walking using a wearable inertia sensing device | Laboratory | 40 | Cross-sectional |
| Lepetit 2018 [86] | Evaluation of the kinetic energy of the torso by magneto-inertial measurement unit during the sit-to-stand movement | Laboratory | 26 | Cross-sectional |
| Levy 2019 [36] | Digital markers of motor activity captured over smartphone is associated with negative symptoms of schizophrenia: Results from a pilot observational study | Laboratory | 30 | Cross-sectional |
| Lipsmeier 2019a [96] | Preliminary reliability and validity of a novel digital biomarker smartphone application to assess cognitive and motor symptoms in Huntington's disease (HD) | NR | 41 | 2 weeks |
| Lipsmeier 2019b [97] | Preliminary validation smartphone application to assess motor symptoms in recently diagnosed Parkinson patients | Laboratory | 113 | 2 weeks |
| Lo 2019 [49] | Predicting motor, cognitive & functional impairment in Parkinson's | Laboratory; Home | 237 | 18 months |
| Lopane 2015 [56] | Dyskinesia detection and monitoring by a single sensor in patients with Parkinson's disease | Laboratory | 82 | Cross-sectional |
| Louter 2015 [67] | Accelerometer-based quantitative analysis of axial nocturnal movements differentiates patients with Parkinson's disease, but not high-risk individuals, from controls | Laboratory | 57 | Cross-sectional |
| Lowes 2019 [104] | Activity monitoring in neuromuscular disease: Successes, challenges, and a path forward | Laboratory; Home | NR | 2 years |
| Ma 2018 [37] | Validation of a Kinect v2 based rehabilitation game | NR | NR | Cross-sectional |
| Mastoras 2019 [92] | Touchscreen typing pattern analysis for remote detection of the depressive tendency | NR | 31 | Cross-sectional |
| Mitsi 2017 [38] | Biometric digital health technology for measuring motor function in Parkinson's disease: Results from a feasibility and patient satisfaction study | Laboratory | 38 | Cross-sectional |
| Montalban 2019 [26] | FLOODLIGHT: Smartphone-Based Self-Monitoring is Accepted by Patients and Provides Meaningful, Continuous Digital Outcomes Augmenting Conventional In-Clinic Multiple Sclerosis Measures | Laboratory; Home | 101 | 24 weeks |
| Ossig 2016 [68] | Correlation of quantitative motor state assessment using a kinetograph and patient diaries in advanced PD: Data from an observational study | Laboratory | 24 | 6 days (considered cross-sectional) |
| Otte 2016 [69] | Accuracy and reliability of the Kinect version 2 for clinical measurement of motor function | NR | 19 | Cross-sectional |
| Ozinga 2017 [39] | Use of mobile device accelerometry to enhance evaluation of postural instability in Parkinson disease | Laboratory | 28 | Cross-sectional |
| Pérez-López 2016 [60] | Assessing motor fluctuations in Parkinson's disease patients based on a single inertial sensor | Laboratory; Home | 15 | Cross-sectional |
| Pradhan 2019 [40] | Quantifying physical activity in early Parkinson disease using a commercial activity monitor | NR | 60 | 14 days |
| Prince 2018 [52] | Big data in Parkinson's disease: Using smartphones to remotely detect longitudinal disease phenotypes | NR | 548 | 6 months |
| Ren 2020 [106] | Multivariate analysis of joint motion data by Kinect: Application to Parkinson's disease | NR | 57 | Cross-sectional |
| Rodríguez-Martín 2017 [61] | A waist-worn inertial measurement unit for long-term monitoring of Parkinson's disease patients | Home | 12 | 30 days |
| Rodríguez-Molinero 2015 [62] | Validation of a portable device for mapping motor and gait disturbances in Parkinson's disease | Laboratory; Home | 35 | Cross-sectional |
| Rodríguez-Molinero 2017 [98] | Analysis of correlation between an accelerometer-based algorithm for detecting Parkinsonian gait and UPDRS subscales | Home | 75 | Cross-sectional |
| Scano 2018 [57] | Kinect v2 implementation and testing of the reaching performance scale for motor evaluation of patients with neurological impairment | Laboratory | 20 | Cross-sectional |
| Seiffert 2019 [109] | Mobile test of manual dexterity in the diagnostics of frailty in older patients with mild Parkinsonian signs | Laboratory | 90 | Cross-sectional |
| Seok 2019 [66] | Quantitative evaluation of hand motor function using a gyrosensor in mild and moderate carpal tunnel syndrome | Laboratory | 97 | Cross-sectional |
| Shaafi Kabiri 2019 [102] | Utility of a smartphone app in discretely assessing and monitoring symptoms of Parkinson's disease | Laboratory; Home | 44 | 9 (Range: 5-13) days |
| Shawen 2019 [108] | Automatic scoring of Parkinson's disease motor symptoms using a smartwatch | Laboratory | 22 | Cross-sectional |
| Sigcha 2020 [112] | Deep learning approaches for detecting freezing of gait in Parkinson's disease patients through on-body acceleration sensors | Home | 21 | Cross-sectional |
| Simonsen 2017 [85] | Design and test of an automated version of the modified Jebsen test of hand function using Microsoft Kinect | Laboratory | 11 | Cross-sectional |
| Taylor-Piliae 2016 [41] | Objective fall risk detection in stroke survivors using wearable sensor technology: A feasibility study | Home | 20 | 3 days |
| Terashi 2020 [71] | Analysis of non-invasive gait recording under free-living conditions in patients with Parkinson's disease: Relationship with global cognitive function and motor abnormalities | Home | 106 | Cross-sectional |
| Terui 2018 [72] | New evaluation of trunk movement and balance during walking in COPD patients by a triaxial accelerometer | Laboratory | 42 | Cross-sectional |
| van Wamelen 2019 [53] | Non-motor correlates of wrist-worn wearable sensor use in Parkinson's disease: An exploratory analysis | Laboratory | 108 | 6 days (considered cross-sectional) |
| Vianello 2017 [58] | Motorbrain: A mobile app for the assessment of users' motor performance in neurology | NR | 133 | Cross-sectional |
| do Carmo Vilas-Boas 2019a [77] | Full-body motion assessment: Concurrent validation of two body tracking depth sensors versus a gold standard system during gait | Laboratory | 20 | Cross-sectional |
| do Carmo Vilas-Boas 2019b [78] | Validation of a single RGB-D camera for gait assessment of polyneuropathy patients | Laboratory | 10 | Cross-sectional |
| Wissel 2017 [101] | Tablet-based application for objective measurement of motor fluctuations in Parkinson disease | Laboratory | 22 | Cross-sectional |
| Zach 2017 [76] | The patient's perspective: The effect of levodopa on Parkinson symptoms | Laboratory | 43 | Cross-sectional |
| Zhan 2018 [115] | Using smartphones and machine learning to quantify Parkinson disease severity: The mobile Parkinson disease score | Home | 250 | 6 months |
| Zhou 2019 [105] | Instrumented trail-making task to identify cognitive-motor impairment and assess cognitive frailty | NR | 54 | Cross-sectional |

Table S9: ROBINS-E risk of bias assessment for primary studies.

| **First Author & Year** | **Confounding** | **Measurement of the Exposure** | **Selection of Participants into the Study or Analysis** | **Post-exposure Interventions** | **Missing Data** | **Measurement of the Outcome** | **Selection of the Reported Result** |
| --- | --- | --- | --- | --- | --- | --- | --- |
| Abrami 2020 [27] | Low risk | Low risk | High risk | Low risk | Low risk | Low risk | Low risk |
| Adams 2015 [28] | Low risk | Low risk | High risk | Low risk | Low risk | Low risk | Low risk |
| Aghanavesi 2020a [80] | Low risk | Low risk | High risk | Low risk | Low risk | Low risk | Low risk |
| Ahlrichs 2016 [93] | High risk | Low risk | High risk | Low risk | Some concerns | Low risk | Low risk |
| Alberts 2015 [29] | Low risk | Low risk | Low risk | Low risk | Low risk | Low risk | Low risk |
| Andrzejewski 2016 [99] | Low risk | Low risk | High risk | Low risk | Low risk | Low risk | Low risk |
| Arora 2018 [42] | Low risk | Low risk | High risk | Low risk | Some concerns | Low risk | Low risk |
| Arroyo-Gallego 2017 [103] | Low risk | Low risk | High risk | Low risk | High risk | Low risk | Low risk |
| Arroyo-Gallego 2018 [59] | Low risk | Low risk | High risk | Low risk | High risk | Low risk | Low risk |
| Beange 2019 [73] | Low risk | Low risk | Low risk | Low risk | Some concerns | Low risk | Low risk |
| Bennasar 2018 [43] | Low risk | Low risk | High risk | Low risk | Some concerns | Low risk | Low risk |
| Bernad-Elazari 2016 [87] | Low risk | Low risk | High risk | Low risk | Some concerns | Low risk | Low risk |
| Bonnechère 2018 [113] | Low risk | Low risk | High risk | Low risk | Some concerns | Low risk | Low risk |
| Boukhvalova 2018 [25] | Low risk | Low risk | High risk | Low risk | Some concerns | Low risk | Low risk |
| Brooks 2020 [30] | Low risk | Low risk | High risk | Low risk | High risk | Low risk | Low risk |
| Campos 2018 [74] | Low risk | Low risk | High risk | Low risk | Low risk | Low risk | Low risk |
| Capecci 2016 [54] | Low risk | Low risk | High risk | Low risk | Some concerns | Low risk | Low risk |
| Cavallo 2019 [55] | Low risk | Low risk | High risk | Low risk | Some concerns | Low risk | Low risk |
| Chae 2020 [63] | Low risk | Low risk | High risk | Low risk | High risk | Low risk | Low risk |
| Christiansen 2017 [31] | Low risk | Low risk | High risk | Low risk | Low risk | Low risk | Low risk |
| de Paula 2018 [84] | High risk | Low risk | High risk | Low risk | Some concerns | Low risk | Low risk |
| Dehbandi 2017 [32] | Low risk | Low risk | Low risk | Low risk | Low risk | Low risk | Low risk |
| Del Din 2018a [45] | Low risk | Low risk | High risk | Low risk | Some concerns | Low risk | Some concerns |
| Del Din 2018b [46] | Low risk | Low risk | High risk | Low risk | Some concerns | Low risk | Some concerns |
| Dowling 2018 [33] | High risk | Low risk | High risk | Low risk | Some concerns | Low risk | Some concerns |
| Evers 2019 [75] | Low risk | Low risk | High risk | Low risk | High risk | Low risk | Some concerns |
| Gordon 2019 [107] | Low risk | Low risk | High risk | Low risk | High risk | Low risk | Low risk |
| Hasan 2019 [47] | Low risk | Low risk | High risk | Low risk | Some concerns | Low risk | Low risk |
| Hiorth 2016 [91] | Low risk | Low risk | High risk | Low risk | Low risk | Low risk | Low risk |
| Horigome 2020 [70] | Low risk | Low risk | High risk | Low risk | Some concerns | Low risk | Low risk |
| Horne 2015 [100] | Low risk | Low risk | High risk | Low risk | High risk | Low risk | Low risk |
| Hssayeni 2018 [114] | Low risk | Low risk | High risk | Low risk | Some concerns | Low risk | Low risk |
| Hssayeni 2019 [34] | High risk | Low risk | High risk | Low risk | Some concerns | Low risk | Low risk |
| Hsu 2019 [81] | Low risk | Low risk | High risk | Low risk | Some concerns | Low risk | Low risk |
| Huang 2019 [82] | Low risk | Low risk | High risk | Low risk | Low risk | Low risk | Low risk |
| Hughes 2019 [35] | Low risk | Low risk | High risk | Low risk | Some concerns | Low risk | Low risk |
| Iakovakis 2018 [88] | Low risk | Low risk | High risk | Low risk | Some concerns | Low risk | Some concerns |
| Kassavetis 2016 [28] | Low risk | Low risk | High risk | Low risk | Some concerns | Low risk | Low risk |
| Kim 2015 [111] | High risk | Low risk | High risk | Low risk | Some concerns | Low risk | Low risk |
| Kim 2018 [64] | High risk | Low risk | High risk | Low risk | Some concerns | Low risk | Low risk |
| Klingelhoefer 2019 [94] | Low risk | Low risk | High risk | Low risk | Some concerns | Low risk | Some concerns |
| Lalvay 2017 [95] | Low risk | Low risk | High risk | Low risk | Low risk | Low risk | Low risk |
| Lauraitis 2020 [90] | Low risk | Low risk | High risk | Low risk | Low risk | Low risk | Low risk |
| Lee 2016a [90] | Low risk | Low risk | High risk | Low risk | Some concerns | Low risk | Low risk |
| Lee 2016b [83] | Low risk | Low risk | High risk | Low risk | Some concerns | Low risk | Low risk |
| Lee 2018 [65] | Low risk | Low risk | High risk | Low risk | High risk | Low risk | Some concerns |
| Lepetit 2018 [86] | Low risk | Low risk | Low risk | Low risk | Some concerns | Low risk | Low risk |
| Levy 2019 [36] | High risk | Low risk | High risk | Low risk | Some concerns | Low risk | Some concerns |
| Lipsmeier 2019a [96] | High risk | Low risk | High risk | Low risk | High risk | Low risk | Some concerns |
| Lipsmeier 2019b [97] | High risk | Low risk | High risk | Low risk | Some concerns | Low risk | Some concerns |
| Lo 2019 [49] | Low risk | Low risk | High risk | Low risk | Low risk | Low risk | Low risk |
| Lopane 2015 [56] | Low risk | Low risk | High risk | Low risk | Some concerns | Low risk | Some concerns |
| Louter 2015 [67] | Low risk | Low risk | High risk | Low risk | High risk | Low risk | Low risk |
| Lowes 2019 [104] | High risk | Low risk | High risk | Low risk | Some concerns | Low risk | Some concerns |
| Ma 2018 [37] | Low risk | Low risk | Low risk | Low risk | Some concerns | Low risk | Low risk |
| Mastoras 2019 [92] | Low risk | Low risk | High risk | Low risk | Some concerns | Low risk | Low risk |
| Mitsi 2017 [38] | Low risk | Low risk | High risk | Low risk | Some concerns | Low risk | Low risk |
| Montalban 2019 [26] | High risk | Low risk | High risk | Low risk | Some concerns | Low risk | Some concerns |
| Ossig 2016 [68] | Low risk | Low risk | High risk | Low risk | High risk | Low risk | Some concerns |
| Otte 2016 [69] | Low risk | Low risk | Low risk | Low risk | Some concerns | Low risk | Low risk |
| Ozinga 2017 [39] | Low risk | Low risk | High risk | Low risk | Low risk | Low risk | Low risk |
| Pantall 2018a [50] | Low risk | Low risk | High risk | Low risk | Low risk | Low risk | Some concerns |
| Pérez-López 2016 [60] | Low risk | Low risk | High risk | Low risk | Some concerns | Low risk | Low risk |
| Pradhan 2019 [40] | Low risk | Low risk | High risk | Low risk | Some concerns | Low risk | Low risk |
| Prince 2018 [52] | Low risk | Low risk | High risk | Low risk | Some concerns | Low risk | Low risk |
| Ren 2020 [106] | Low risk | Low risk | High risk | Low risk | Some concerns | Low risk | Low risk |
| Rodríguez-Martín 2017 [61] | Low risk | Low risk | High risk | Low risk | Some concerns | Low risk | Some concerns |
| Rodríguez-Molinero 2015 [62] | High risk | Low risk | High risk | Low risk | High risk | Low risk | Some concerns |
| Rodríguez-Molinero 2017 [98] | Low risk | Low risk | High risk | Low risk | Some concerns | Low risk | Low risk |
| Scano 2018 [57] | Low risk | Low risk | High risk | Low risk | Low risk | Low risk | Low risk |
| Seiffert 2019 [109] | High risk | Low risk | High risk | Low risk | Some concerns | Low risk | Some concerns |
| Seok 2019 [66] | Low risk | Low risk | High risk | Low risk | Some concerns | Low risk | Low risk |
| Shaafi Kabiri 2019 [102] | Low risk | Low risk | High risk | Low risk | Low risk | Low risk | Some concerns |
| Shawen 2019 [108] | High risk | Low risk | High risk | Low risk | Some concerns | Low risk | Some concerns |
| Sigcha 2020 [112] | Low risk | Low risk | High risk | Low risk | Some concerns | Low risk | Low risk |
| Simonsen 2017 [85] | Low risk | Low risk | High risk | Low risk | Some concerns | Low risk | Low risk |
| Taylor-Piliae 2016 [41] | Low risk | Low risk | High risk | Low risk | Some concerns | Low risk | Low risk |
| Terashi 2020 [71] | Low risk | Low risk | High risk | Low risk | Some concerns | Low risk | Low risk |
| Terui 2018 [72] | Low risk | Low risk | High risk | Low risk | Some concerns | Low risk | Low risk |
| van Wamelen 2019 [53] | Low risk | Low risk | High risk | Low risk | Low risk | Low risk | Low risk |
| Vianello 2017 [58] | Low risk | Low risk | Low risk | Low risk | Some concerns | Low risk | Low risk |
| do Carmo Vilas-Boas 2019a [77] | Low risk | Low risk | Low risk | Low risk | Some concerns | Low risk | Low risk |
| do Carmo Vilas-Boas 2019b [78] | Low risk | Low risk | High risk | Low risk | Some concerns | Low risk | Low risk |
| Wissel 2017 [101] | Low risk | Low risk | High risk | Low risk | Some concerns | Low risk | Low risk |
| Zach 2017 [76] | Low risk | Low risk | High risk | Low risk | Low risk | Low risk | Low risk |
| Zhan 2018 [115] | Low risk | Low risk | High risk | Low risk | High risk | Low risk | Low risk |
| Zhou 2019 [105] | Low risk | Low risk | High risk | Low risk | Some concerns | Low risk | Some concerns |

Table S10: Validity criteria of the technologies investigated by the included publications.

| **Type of Validation Used** | **Validation, Longitudinal or Cross-sectional^a^** | **Technology or Device Name** | **Author & Year^b^** |
| --- | --- | --- | --- |
| Clinical condition | Cross-sectional | Activinsights | Bennasar 2018 [43] |
| Clinical condition | Cross-sectional | AnySoftKeyboard | Arroyo-Gallego 2017 [103] |
| Clinical condition | Cross-sectional | Automated, infrared-assisted, trunk accelerometer-based gait analysis system | Hsu 2019 [81] |
| Clinical condition | Cross-sectional | Dynaport Sensor | Louter 2015 [67] |
| Clinical condition | Cross-sectional | iMotor tablet application | Mitsi 2017 [38] |
| Clinical condition | Cross-sectional | Inertial sensor | Lee 2018 [65] |
| Clinical condition | Cross-sectional | Mememtum | Lalvay 2017 [95] |
| Clinical condition | Cross-sectional | Microsoft Kinect 2 (MK2) | Horigome 2020 [70] |
| Clinical condition | Cross-sectional | Microsoft Kinect 2 (MK2) | Ren 2020 [106] |
| Clinical condition | Cross-sectional | Microsoft Kinect 2 (MK2) | Bonnechère 2018 [113] |
| Clinical condition | Cross-sectional | Miniature and light-weight gyrosensor (6 × 10 × 25 mm, 0.26 g, CG-L53; NEC/Tokin) | Seok 2019 [66] |
| Clinical condition | Cross-sectional | MotorBrain | Vianello 2017 [58] |
| Clinical condition | Cross-sectional | Neural Impairment Test Suite (NITS) | Lauraitis 2020 [90] |
| Clinical condition | Cross-sectional | Nokia 500 model application | de Paula 2018 [84] |
| Clinical condition | Cross-sectional | outREACH sensor | Hughes 2019 [35] |
| Clinical condition | Cross-sectional | SensHand V1 | Cavallo 2019 [55] |
| Clinical condition | Cross-sectional | Sensors on wrists, ankles, lower back, and in the front trouser pocket | Evers 2019 [75] |
| Clinical condition | Cross-sectional | Smartphone tapper (SmT) application | Lee 2016a [90] |
| Clinical condition | Cross-sectional | Smartphone-based passive assessment | Levy 2019 [36] |
| Clinical condition | Cross-sectional | Tablet application | Huang 2019 [82] |
| Clinical condition | Cross-sectional | Tablet application | Seiffert 2019 [109] |
| Clinical condition | Cross-sectional | TapPD application | Hasan 2019 [47] |
| Clinical condition | Longitudinal | ActiGraph GT3X [+ and BT] | Christiansen 2017 [31] |
| Clinical condition | Longitudinal | activPAL3 | Hiorth 2016 [91] |
| Clinical condition | Longitudinal | Axivity AX3 | Pantall 2018a [50] |
| Clinical condition | Longitudinal | Axivity AX3 | Pantall 2018b [51] |
| Clinical condition | Longitudinal | Axivity AX3 | Coates 2020 [44] |
| Clinical condition | Longitudinal | Dynaport Hybrid | Bernad-Elazari 2016 [87] |
| Clinical condition | Longitudinal | Fitbit | Pradhan and Kelly 2019 [40] |
| Clinical condition | Longitudinal | Inertial sensor | Pérez-López 2016 [60] |
| Clinical condition | Longitudinal | Lenovo G50-70 i3-4005U with 4GB of memory and a 15-inch screen running Manjaro Linux operative system | Arroyo-Gallego 2018 [59] |
| Clinical condition | Longitudinal | mPower iPhone application | Prince 2018 [52] |
| Clinical condition | Longitudinal | PAMSys-X™ sensors | Andrzejewski 2016 [99] |
| Clinical condition | Longitudinal | PAMSys-X™ sensors | Taylor-Piliae 2016 [41] |
| Clinical condition | Longitudinal | Parkinson’s KinetiGraph™ (PKG) | Horne 2015 [100] |
| Clinical condition | Longitudinal | Smartphone application | Arora 2018 [42] |
| Clinical condition | Longitudinal | Watch style W270, LG | Chae 2020 [63] |
| Clinical condition | Longitudinal | Wearable device placed on the lower back | Del Din 2018a [45] |
| Clinical condition | Longitudinal | Wearable device placed on the lower back | Del Din 2018b [46] |
| Clinician rating | Cross-sectional | Android smartphone (Nexus 5; Google, Inc.) application | Kim 2015 [111] |
| Clinician rating | Cross-sectional | Android smartphone (Nexus 5; Google, Inc.) application | Kim 2018 [64] |
| Clinician rating | Cross-sectional | Ankle motion sensors (3-axial accelerometers and gyroscopes) | Aghanavesi 2020b [79] |
| Clinician rating | Cross-sectional | Smartphone application | Capecci 2016 [54] |
| Clinician rating | Cross-sectional | Waist-mounted sensor | Ahlrichs 2016 [93] |
| Clinician rating | Longitudinal | Inertial sensor | Rodríguez-Martín 2017 [61] |
| Clinician rating | Longitudinal | Smartphone application | Boukhvalova 2018 [25] |
| Patient-reported | Longitudinal | Parkinson’s KinetiGraph™ (PKG) | Ossig 2016 [68] |
| Research device | Cross-sectional | ActiGraph physical monitoring devices | Campos 2018 [74] |
| Research device | Cross-sectional | iPad | Alberts 2015 [29] |
| Research device | Cross-sectional | iPad | Ozinga 2017 [39] |
| Research device | Cross-sectional | MetaMotionR IMUs | Beange 2019 [73] |
| Research device | Cross-sectional | Microsoft Kinect 2 (MK2) | Ma 2018 [37] |
| Research device | Cross-sectional | Microsoft Kinect 2 (MK2) | Otte 2016 [69] |
| Research device | Cross-sectional | Microsoft Kinect 2 (MK2) | do Carmo Vilas-Boas 2019a [77] |
| Research device | Cross-sectional | Microsoft Kinect 2 (MK2) | do Carmo Vilas-Boas 2019b [78] |
| Research device | Cross-sectional | MIMU (APDM, Opal sensor) | Lepetit 2018 [86] |
| Traditional standard tests | Cross-sectional | Android smartphone (LG Nexus 5X) typing application; iPrognosis | Iakovakis 2018 [88] |
| Traditional standard tests | Longitudinal | Android smartphone (LG Nexus 5X) typing application; iPrognosis | Iakovakis 2019 [89] |
| Traditional standard tests | Cross-sectional | HTC Desire smartphone application | Kassavetis 2016 [48] |
| Traditional standard tests | Cross-sectional | iMotor tablet application | Wissel 2017 [101] |
| Traditional standard tests | Cross-sectional | Inertial sensor | Lopane 2015 [56] |
| Traditional standard tests | Cross-sectional | Inertial sensor | Rodríguez-Molinero 2017 [98] |
| Traditional standard tests | Cross-sectional | Inertial sensor | Rodríguez-Molinero 2015 [62] |
| Traditional standard tests | Cross-sectional | Instrumented trail-making task (iTMT) on a wearable sensor | Zhou 2019 [105] |
| Traditional standard tests | Cross-sectional | Lightweight biaxial piezoelectric accelerometer (Medifactory international) | Zach 2017 [76] |
| Traditional standard tests | Cross-sectional | Microsoft Kinect 2 (MK2) | Dehbandi 2017 [32] |
| Traditional standard tests | Cross-sectional | Microsoft Kinect 2 (MK2) | Scano 2018 [57] |
| Traditional standard tests | Cross-sectional | Microsoft Kinect 2 (MK2) | Simonsen 2017 [85] |
| Traditional standard tests | Cross-sectional | MIMAMORI-Gait | Terashi 2020 [71] |
| Traditional standard tests | Cross-sectional | Opal devices (APDM Wearable Technologies) | Abrami 2020 [27] |
| Traditional standard tests | Cross-sectional | Shimmer3 IMUs | Aghanavesi 2020a [80] |
| Traditional standard tests | Cross-sectional | Smartphone application | Lo 2019 [49] |
| Traditional standard tests | Cross-sectional | Smartwatch | Shawen 2019 [108] |
| Traditional standard tests | Cross-sectional | Triaxial accelerometer | Sigcha 2020 [112] |
| Traditional standard tests | Cross-sectional | Wireless triaxial accelerometer (MG-M1110; LSI Medience) | Terui 2018 [72] |
| Traditional standard tests | Longitudinal | Fitbit Flex 2 | Lowes 2019 [104] |
| Traditional standard tests | Longitudinal | Floodlight Smartphone application | Montalban 2019 [26] |
| Traditional standard tests | Longitudinal | iPrognosis Application | Klingelhoefer 2019 [94] |
| Traditional standard tests | Longitudinal | Microsoft Kinect 2 (MK2) | Adams 2015 [28] |
| Traditional standard tests | Longitudinal | Parkinson’s KinetiGraph™ (PKG) | van Wamelen 2019 [53] |
| Traditional standard tests | Longitudinal | Philips Actiwatch | Brooks 2020 [30] |
| Traditional standard tests | Longitudinal | Smartphone application | Lipsmeier 2019a [96] |
| Traditional standard tests | Longitudinal | Smartphone application | Lipsmeier 2019b [97] |
| Traditional standard tests | Longitudinal | Smartphone application | Zhan 2018 [115] |
| Traditional standard tests | Longitudinal | Smartphone application that quantitatively measures hand dexterity | Lee 2016b [83] |
| Traditional standard tests | Longitudinal | Smartphone or smart watch application | Gordon 2019 [107] |
| Traditional standard tests | Longitudinal | Smartphone or smart watch application | Shaafi Kabiri 2019 [102] |
| Traditional standard tests | Longitudinal | TypeOfMood | Mastoras 2019 [92] |
| Treatment status | Cross-sectional | APDM wearable technology | Dowling 2018 [33] |
| Treatment status | Cross-sectional | KinetiSense | Hssayeni 2019 [34] |
| Treatment status | Cross-sectional | Triaxial gyroscope sensor | Hssayeni 2018 [114] |

^a^Whether the validation procedures were cross-sectional (single timepoint) or longitudinal (multiple timepoints or over time).

^b^References with “a” at the end are the first alphabetical title in a group of publications with the same first author and year.

Figure S1: Funnel plot.


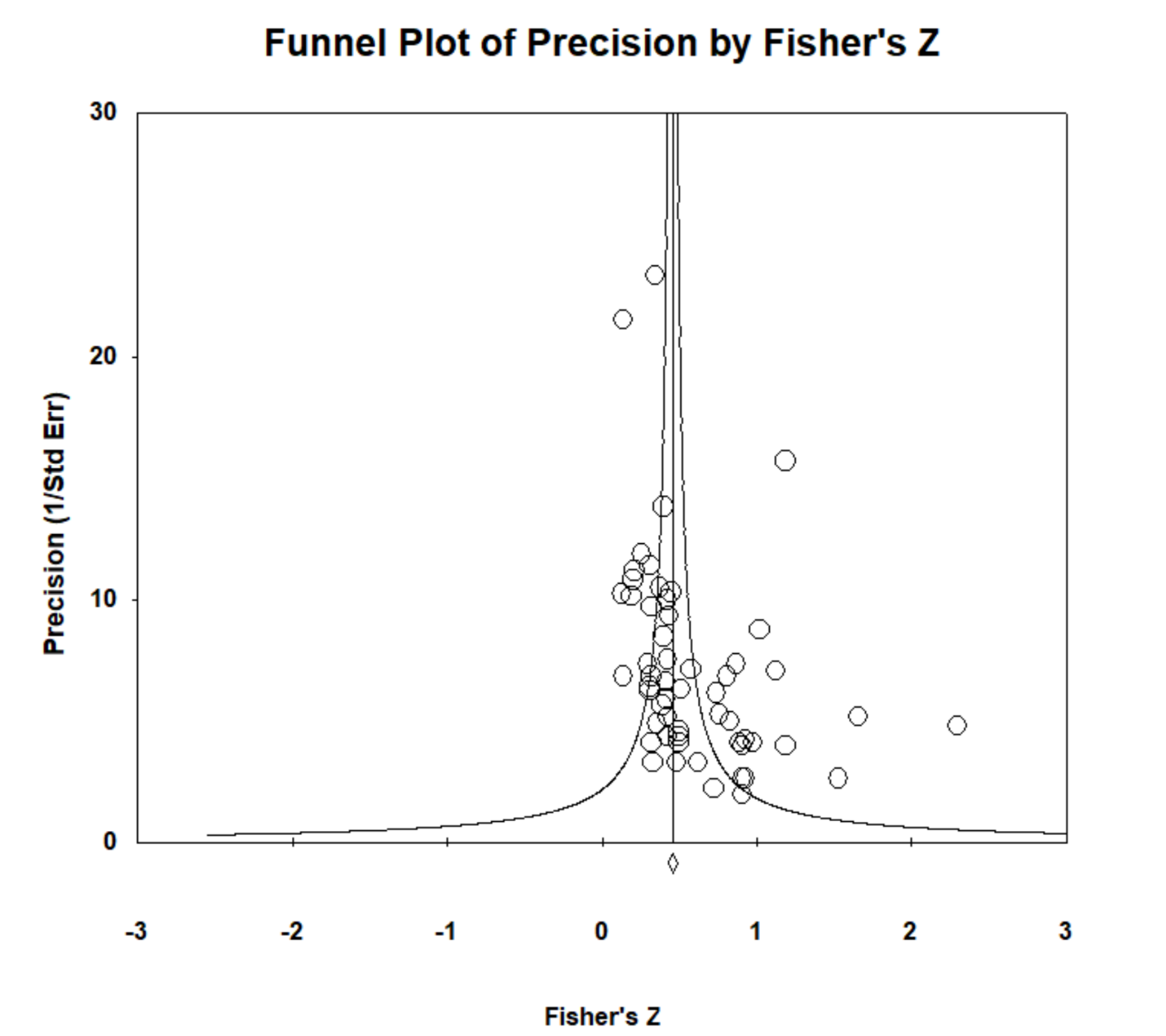

Supplement: Multimedia Appendix 1 [file jmir_v24i11e37683_app1.docx]
